# Supplementary material for: Why do children attend school, engage in other activities or socialise when they have symptoms of an infectious illness? A cross-sectional survey
Source: BMJ Open. 2023 Nov 17;13(11):e071599. doi: 10.1136/bmjopen-2023-071599 (PMC10660639; doi:10.1136/bmjopen-2023-071599)
Supplement: Supplementary data [file bmjopen-2023-071599supp001.pdf]

Covid-19 Trust & Perceptions

November-December 2021

Text S1: Survey questions

Standard demographic variables:

1. What is your date of birth?

1. YEAR
2. 1910 1910
3. ...
4. 2015 2015
5. MONTH
6. January
7. February
8. March
9. April
10. May
11. June
12. July
13. August
14. September
15. October
16. November
17. December

2. Which of the following describes how you think of yourself?

1. Male
2. Female
3. In another way
4. Prefer not to answer

3. Where do you live? Please note: This question may be considered personal. We would like to remind you that your participation is strictly voluntary and that your responses are used for research purposes only. The answers that you provide will be presented in aggregate form and none of them will be linked back to you in any way. All data will be collected and processed in adherence to the Market Research Society's Code of Conduct and the General Data Protection Regulation (GDPR).

1. Postcode
2. Postal Town
3. Prefer Not to Answer

4. Where do you live?

1. North East
2. North West
3. Yorkshire and The Humber
4. West Midlands
5. East Midlands
6. East of England

Woodland L, *et al. BMJ Open* 2023; 13:e071599. doi: 10.1136/bmjopen-2023-071599

7. South West
8. South East
9. Greater London
10. Wales
11. Scotland
12. Northern Ireland

5. How many people are living or staying at your current address? (Include yourself and any other adults or children who are currently living or staying at this address for at least two months)

1. 1
2. 2
3. 3
4. 4
5. 5
6. 6
7. 7
8. 8
9. 9
10. 10
11. 11
12. 12+

6. How many children under the age of 18 are living in your household? Please reference only the children for which you are the parent or legal guardian. (If there are no children under 18 in your household, please type 0)

7. Please provide us with the following information about the children under the age of 18 in your household. Please reference only the children for which you are the parent or legal guardian.

1. Relationship
2. Parent (biological or adopted)
3. Legal guardian
4. Other (e.g. step-child)
5. Gender
6. Boy
7. Girl
8. Year of birth
9. Month of birth

8. What is your highest level of education attained?

1. Primary school
2. Secondary school (age under 15 years old)
3. GNVQ / GSVQ / GCSE/ SCE standard.
4. NVQ1, NVQ2
5. NVQ3/ SCE Higher Grade/ Advanced GNVQ/ GCE A/AS or similar.
6. NVQ4 / HNC / HND / Bachelor's degree or similar.
7. NVQ5 or post-graduate diploma.

Covid-19 Trust & Perceptions

November-December 2021

9. The next question may be considered personal, but it is not mandatory to answer. If you do, we assure you that your responses will be kept strictly confidential and used for research purposes only. What is the COMBINED TOTAL ANNUAL INCOME (pre-tax) earned by all members of your household? Please include all your income sources : salaries, scholarships, pension and Social Security benefits, dividends from shares, income from rental properties, child support and alimony etc.

1. Under £5,000
2. £5,000 - £9,999
3. £10,000 - £14,999
4. £15,000 - £19,999
5. £20,000 - £24,999
6. £25,000 - £34,999
7. £35,000 - £44,999
8. £45,000 - £54,999
9. £55,000 - £99,999
10. £100,000 or more
11. Prefer not to answer

10. What is your current employment status?

1. Employed full-time
2. Employed part-time
3. Self employed
4. Unemployed but looking for a job
5. Unemployed and not looking for a job/Long-term sick or disabled
6. Full-time parent, homemaker
7. Retired
8. Student/Pupil

11. In which of the below categories does your occupation fall? If retired or unemployed, please indicate the category closest to your previous occupation.

Responses copied from https://www.ilo.org/public/english/bureau/stat/isco/isco88/

12. What is the occupation of the person with the highest income? If retired or unemployed, please indicate the category closest to his/her previous occupation.

Responses copied from https://www.ilo.org/public/english/bureau/stat/isco/isco88/

In this survey, some questions will be about health and perceptions about, coronavirus, politics, religious beliefs, etc. A "Prefer not to answer" option will be available for you to select, if the case. Ipsos is running the survey and collecting such data in order to understand attitudes towards public health measures in the context of the coronavirus pandemic, and trust in their efficacy.

Ipsos has been commissioned by the University of Bristol and King's College London to carry out this research. Information collected through the survey will be made available to the team of researchers at the University of Bristol and King's College London in an anonymised format, unless you provide your permission to pass personal data to them. At

Woodland L, *et al. BMJ Open* 2023; 13:e071599. doi: 10.1136/bmjopen-2023-071599

no point will any information that would allow you to be identified be made available to the public.

Participation is completely voluntary and you may withdraw your consent at any time. Your survey answers will be combined with the answers from all other participants and used for social research purposes only, and your personal data will be held for no longer than 12 months within Ipsos and no longer than April 2023 from researchers at the University of Bristol and King's College London.

Do you accept the collection of personal information and of health and perceptions about coronavirus related data?

1. *Yes, I accept*
2. *No, I do not accept*

13. Which of these applies to your home?

1. Being bought on a mortgage
2. Owned outright by household
3. Rented from a local authority
4. Rented from a housing association/trust
5. Rented from a private landlord
6. Other
7. Refused/don't know

The next question may be considered personal, but it is not mandatory to answer. If you do, we assure you that your responses will be kept strictly confidential and used for research purposes only.

14. What is your ethnic group?

Please select only one

White

1. English / Welsh / Scottish / Northern Irish / British
2. Irish
3. Gypsy or Irish Traveller
4. Any other White background

Mixed / multiple ethnic groups

5. White and Black Caribbean
6. White and Black African
7. White and Asian
8. Any other Mixed / multiple ethnic background

Asian / Asian British

9. Indian
10. Pakistani

11. Bangladeshi
12. Chinese
13. Any other Asian background

Black / African / Caribbean / Black British

14. African
15. Caribbean
16. Any other Black / African / Caribbean background

Other ethnic group

17. Arab
18. Any other ethnic group, please write in
19. Prefer not to answer

Survey questions

For the next set of questions, we would like you to think about your [number of children] youngest children who usually attend school.

15. Since *about* [Scotland only] the start of the school year (September 2021), please tick, for each child, any new symptoms that they developed for any reason (e.g. a cold, allergic reaction, coronavirus, etc.), even if some of the symptoms were mild. If they have developed new symptoms more than once during that period (for example if they have had a cold in one week, and an allergic reaction a couple of weeks later), please just tell us about the most severe set of symptoms that they had (for example, tell us about the cold, or the allergic reaction, whichever seemed worse). Please tick any that apply.

1. New, continuous cough
2. High temperature / fever
3. Diarrhoea
4. Nausea / feeling sick
5. Vomiting / being sick
6. Earache
7. A rash
8. Loss of sense of smell (fully or partial)
9. Loss of taste
10. Sore throat
11. Headache
12. Muscle ache
13. Breathlessness, tight chest, or wheezing
14. Feeling tired or exhausted
15. I don't have any (more) children who usually attend school
16. None of these
17. Don't know
18. Prefer not to say

1. Child 1 (youngest)
2. Child 2
3. Child 3
4. Child (oldest)

16. Please think about your child who most recently developed symptoms. When they had symptoms, did they do any of the following?
Please tick any that apply. Do not count things that they did online.

1. Went to school
2. Went to a club or lesson outside of school
3. Visited someone from another household
4. Someone from another household visited the child
5. Someone from another household visited our household
6. Took a lateral flow test (LFT) to see whether they had coronavirus (this is a rapid test that gives results in 30 minutes)
7. Took a polymerase chain reaction (PCR) test to see whether they had coronavirus (this is a test that is sent off to a laboratory)
8. Took both a lateral flow test (LFT, a rapid test that gives results in 30 minutes) and a polymerase chain reaction (PCR) test (a test that you send off to the laboratory) to see whether they had coronavirus
9. Took a test to see whether they had coronavirus, but I am not sure what type of test it was
10. None of these
11. Prefer not to say

17. Please still think about your child who most recently developed symptoms. You said your child had taken at least one rapid lateral flow test (LFT). Were any of the results positive?

1. Yes, this child had one or more rapid lateral flow tests (LFT) and had a positive result
2. None of the rapid lateral flow tests (LFT) for this child had a positive result
3. Don't know
4. Prefer not to say

18. Please still think about your child who most recently developed symptoms. You said your child had taken a polymerase chain reaction (PCR) test to see whether they had coronavirus. What was the result?

1. My child tested positive
2. My child tested negative
3. The result was inconclusive
4. I have not received the results
5. Don't know
6. Prefer not to say

Covid-19 Trust & Perceptions

November-December 2021

19. Thinking about the same child who most recently developed symptoms, to what extent, if at all, do you agree or disagree with the following statements about this child, as a result of having these symptoms?

1. My child has missed too much school since September this year
2. My child is behind at school
3. My child often says they have symptoms of illnesses when they do not
4. Often no-one is available to look after my child if they cannot go to school
5. My child does not want to take time off school
6. My child makes their own decisions about when they go to school
7. My child should go to school if they have taken medication (e.g., Calpol, paracetamol)

    1. Strongly agree
    2. Tend to agree
    3. Neither agree nor disagree
    4. Tend to disagree
    5. Strongly disagree
    6. Don't know
    7. Prefer not to say

20. Now thinking in general, to what extent, if at all, do you agree or disagree with the following statements?

1. If children have common illnesses (e.g., a cold), they should go to school
2. Children build up their immunity by mixing with children who have common illnesses (e.g., a cold)
3. Other children with common illnesses (e.g. a cold) go to school
4. If children have mild symptoms of an illness, they should go to school
5. Going to school is important for my child's mental health
6. When my child says they are too ill to attend school, I let them stay at home

    1. Strongly agree
    2. Tend to agree
    3. Neither agree nor disagree
    4. Tend to disagree
    5. Strongly disagree
    6. Don't know
    7. Prefer not to say

Woodland L, *et al. BMJ Open* 2023; 13:e071599. doi: 10.1136/bmjopen-2023-071599
