# Supplementary material for: Why do children attend school, engage in other activities or socialise when they have symptoms of an infectious illness? A cross-sectional survey
Source: BMJ Open. 2023 Nov 17;13(11):e071599. doi: 10.1136/bmjopen-2023-071599 (PMC10660639; doi:10.1136/bmjopen-2023-071599)
Supplement: Author's manuscript [file bmjopen-2023-071599.draft_revisions.pdf]

BMJ Open

BMJ Open is committed to open peer review. As part of this commitment we make the peer review history of every article we publish publicly available.

When an article is published we post the peer reviewers' comments and the authors' responses online. We also post the versions of the paper that were used during peer review. These are the versions that the peer review comments apply to.

The versions of the paper that follow are the versions that were submitted during the peer review process. They are not the versions of record or the final published versions. They should not be cited or distributed as the published version of this manuscript.

BMJ Open is an open access journal and the full, final, typeset and author-corrected version of record of the manuscript is available on our site with no access controls, subscription charges or pay-per-view fees (http://bmjopen.bmj.com).

If you have any questions on BMJ Open's open peer review process please email info.bmjopen@bmj.com

BMJ Open

Why do children attend school and socialise when they have symptoms of an infectious illness? A cross-sectional survey

Journal:	*BMJ Open*
Manuscript ID	bmjopen-2023-071599
Article Type:	Original research
Date Submitted by the Author:	04-Jan-2023

Complete List of Authors:	Woodland, Lisa; King's College London, Psychological Medicine<br>Smith, Louise; King's College London, Psychological Medicine<br>Webster, Rebecca; The University of Sheffield, Psychology<br>Amlôt, Richard; UK Health Security Agency<br>Rubin, G James; King's College London, Psychological Medicine

Keywords:	INFECTIOUS DISEASES, COVID-19, Health policy < HEALTH SERVICES ADMINISTRATION & MANAGEMENT

SCHOLARONE™
Manuscripts

I, the Submitting Author has the right to grant and does grant on behalf of all authors of the Work (as defined in the below author licence), an exclusive licence and/or a non-exclusive licence for contributions from authors who are: i) UK Crown employees; ii) where BMJ has agreed a CC-BY licence shall apply, and/or iii) in accordance with the terms applicable for US Federal Government officers or employees acting as part of their official duties; on a worldwide, perpetual, irrevocable, royalty-free basis to BMJ Publishing Group Ltd ("BMJ") its licensees and where the relevant Journal is co-owned by BMJ to the co-owners of the Journal, to publish the Work in this journal and any other BMJ products and to exploit all rights, as set out in our licence.

The Submitting Author accepts and understands that any supply made under these terms is made by BMJ to the Submitting Author unless you are acting as an employee on behalf of your employer or a postgraduate student of an affiliated institution which is paying any applicable article publishing charge ("APC") for Open Access articles. Where the Submitting Author wishes to make the Work available on an Open Access basis (and intends to pay the relevant APC), the terms of reuse of such Open Access shall be governed by a Creative Commons licence – details of these licences and which Creative Commons licence will apply to this Work are set out in our licence referred to above.

Other than as permitted in any relevant BMJ Author's Self Archiving Policies, I confirm this Work has not been accepted for publication elsewhere, is not being considered for publication elsewhere and does not duplicate material already published. I confirm all authors consent to publication of this Work and authorise the granting of this licence.

For peer review only

Why do children attend school and socialise when they have symptoms of an infectious illness? A cross-sectional survey

Lisa Woodland MSc,[1,4*] Weston Education Centre, Cutcombe Road, London SE5 9RJ, UK (PhD Researcher in Psychological Medicine)

Louise E Smith PhD,[1,4] Weston Education Centre, Cutcombe Road, London SE5 9RJ, UK (Research Associate in Psychological Medicine)

Rebecca K Webster PhD,[2] Cathedral Court, 1 Vicar Lane, Sheffield, S1 2LT, UK (Lecturer, Department of Psychology)

Richard Amlôt PhD,[3,4] Behavioural Science and Insights Unit (BSIU), Porton Down, Salisbury, SP4 0JG, UK (Deputy Director, Behavioural Science and Insights Unit (BSIU))

G James Rubin PhD,[1,4] Weston Education Centre, Cutcombe Road, London SE5 9RJ, UK (Professor of Psychology & Emerging Health Risks)

[1] King's College London

[2] University of Sheffield

[3] UK Health Security Agency

[4] NIHR Health Protection Research Unit in Emergency Preparedness and Response

Correspondence to: Lisa Woodland, King's College London, Weston Education Centre, Cutcombe Road, London SE5 9RJ, UK, lisa.woodland@kcl.ac.uk

Word Count: 2,645

Key words: Infectious diseases; schools; children; presenteeism; COVID-19; diarrhoea; vomiting

For peer review only

For peer review only - http://bmjopen.bmj.com/site/about/guidelines.xhtml

ABSTRACT

Objectives

To prevent the spread of infectious disease, children are typically asked not to attend school or socialise with others whilst they have specific symptoms. Despite this, many children still attend school whilst symptomatic.

Design and setting

We commissioned a national cross-sectional survey with data collected between 19 November and 18 December 2021.

Participants

Parents (n=941) were asked if their children (aged between four and 17 years), had experienced symptoms since the start of the school year (September 2021), and what, if any, activities that child had done while symptomatic.

Outcome measures

Children who had “engaged in risky behaviour,” (e.g., attended school or socialised whilst symptomatic with stay-at-home symptoms of illness).

Results

We identified 251 parents who had at least one child that exhibited either recent vomiting, diarrhoea, high temperature/fever, a new continuous cough, a loss or change to their sense of taste or smell in the absence of a negative (PCR) COVID-19 test. According to UK Government guidance, these children should have remained at home, yet 33% (n=84, 95% CI 28% to 39%) had attended school or other activities outside the home or socialised with members of another household. Children were significantly more likely to engage in these risky behaviours when parents: were aged 45 and younger; allowed their children to make their own decisions about school attendance; agreed that their child should go to school if they took over the counter medication; or believed that children should go to school if they have mild symptoms of illness.

Conclusion

To reduce the likelihood of children with symptoms of an infectious illness having contact with others, parents and children require clearer advice on the symptoms that indicate a potentially infectious illness and which means that a child should not attend school, engage in activities outside school, or socialise with others.

STRENGTHS AND LIMITATIONS OF THIS STUDY

- A strength of the study is that we investigated reported behaviour, rather than intentions.

For peer review only

- A range of common childhood signs and symptoms of illness are included, such as vomiting, high temperature and symptoms of COVID-19. Therefore, this study is generalisable to illness within schools pre-, during and post-pandemic.
- Data about children's demographics were not collected, which limits the study's findings.

For peer review only

INTRODUCTION

In order to reduce the spread of infectious illness within schools, children who have specific symptoms, including fever, diarrhoea and vomiting, are commonly advised to remain at home [1,2]. While this has been the case for many years, this message was made more urgent by the COVID-19 pandemic. In the UK, throughout most of 2020 and 2021, anyone with a new continuous cough, a high temperature, or a loss or change to their sense of taste or smell were asked not to attend school or work nor to interact with people outside their household unless they had a negative COVID-19 polymerase chain reaction (PCR) test result [3,4]. This message is also important for other symptoms of infectious illnesses. For instance, in February 2022, cases of norovirus were 48% higher than expected in educational settings [5], resulting in a warning to the public about the health threat [5]. Children are particularly susceptible to infections because their immune systems are immature, they are often in close contact with other children, and they have a poor understanding of hygiene practices [2,6]. Preventing ill children from mixing with others is essential in mitigating the spread of infection [7,8]. The UK Government has issued guidance on how long a child should not attend school for when they show symptoms of an infectious illness [2].

Research indicates that a worrying proportion of symptomatic children may be attending school (known as "school-based presenteeism"). One survey of 3,040 secondary school pupils in Norway reported that 58% had attended school in the past year despite feeling so ill that they should have stayed at home [9]. One in six British parents reported that they would send their child to school even if they were currently experiencing diarrhoea or vomiting [10]. A systematic review has suggested that people are more likely to attend school or work with symptoms of an infectious illness if they: are unsure of the sickness guidelines; are worried about disciplinary action; are unable to find alternative child care; are concerned about their workload; feel they have missed too much work; perceive a culture of presenteeism in the organisation; and perceive their illness to be mild or non-infectious [11].

In this study, we used a cross-sectional national survey to identify a sample of children (via their parents) who had recently experienced symptoms that should require them to remain at home. We investigated the proportion of those children who engaged in risky behaviours (e.g., attended school, clubs, or other activities or who socialised with people outside their household). We also investigated a range of possible parent and child-level risk factors for engaging in these activities.

METHODS

Design

As part of a wider piece of work, we commissioned Ipsos MORI [12] to conduct a national cross-sectional online survey of 5,000 participants. Data were collected between 19 November and 18 December 2021.

Participants

Participants aged between 18 and 75, and who lived in the UK were eligible for the survey. We used non-probability sampling with quotas set on age interlocked by

For peer review only - http://bmjopen.bmj.com/site/about/guidelines.xhtml

gender, government office region, working status and social grade. One percent of participants were removed due to Ipsos MORI's quality control procedures. The survey company perform automated quality checks, such as country geo-IP validation and removing duplicate email identifications. Further checks were made on the final data set, and data that indicated inconsistencies, such as straight-lining, when the participant selects the same response throughout the survey, and incomplete surveys were removed. Participants were recruited from a pre-existing pool of potential respondents who had already expressed an interest in receiving market research surveys. Participants were also recruited by advertising via platforms such as socials, online gaming sites and news outlets. Participants were paid between €1 and €1.50 for completing the survey.

4,962 participants completed the survey, of whom 941 indicated that they had a child aged between four and 17 years. Of these, 251 reported that their child had experienced symptom(s) that required them to stay at home.

Patient and public involvement

This study had no patient and public involvement.

Study materials

The wording of all survey items is available in the supplementary materials (Text S1).

Participant demographics

We asked parents to report their gender, age, postcode (to derive region), household income, employment status, ethnicity, and level of education. A non-response option was available for each of these demographic questions ("prefer not to say," "prefer not to answer," and for gender only, parents could also respond "in another way"). Due to space limitations in the survey, we did not collect child demographic data.

Identification of children with symptoms of illness

Parents were asked to consider their four (or fewer) youngest children aged between four and 17 years and to report for each child any symptoms they had experienced since the start of the school year (September 2021). We restricted the number of children parents could report on to four to allow most parents to report the symptoms for all of their children whilst limiting the length of the survey. In Scotland, the academic year started in August 2021, therefore, participants in Scotland were asked about their child's symptoms since "*about* the start of the school year (September 2021)". If a child had experienced multiple bouts of illness during that period, we asked parents to report the one set of symptoms they perceived as most severe. Parents were asked to report all symptoms that applied to their child from a list of 14 symptoms of infectious illnesses and ailments common in children [2].

Children who engaged in activities whilst symptomatic

Parents who had at least one symptomatic child were asked to consider the child who most recently exhibited symptoms and to report whether, when they had symptoms, they engaged in any of the following activities (excluding online): going to school; going to a club or lesson outside of school; visiting someone from another

For peer review only - http://bmjopen.bmj.com/site/about/guidelines.xhtml

household; having someone from another household visit them; having someone from another household visit the household in general. Parents were asked to “tick any [activity] that applied,” or they could respond “none of these” or “prefer not to say.”

We also asked parents whether their child had taken a COVID-19 test (lateral flow (LFT) test or a PCR test) while symptomatic, and if so, the result of the test.

Parent attitudes about their child and perceptions about illness

We asked parents 13 statements about their attitudes concerning their child (e.g., my child has missed too much school since September this year) and about common perceptions about illness (e.g., other children with common illnesses (e.g., a cold) go to school). We asked parents to respond to each statement on a five-point Likert scale from “strongly agree” to “strongly disagree”; they could also respond “don’t know” or “prefer not to say.”

Ethics

The research was approved by the Faculty of Social Sciences and Law, University of Bristol (approval code 116976 and 8273). Participants provided informed consent.

Reporting

We used the STROBE cross sectional checklist when writing our report [13].

Analysis

We created binary and multinominal variables for parent demographics (presented in Table 1).

We recoded symptoms that necessitated the child to “stay-at-home” according to Government guidance as being: a new, continuous cough or a loss or change to their sense of taste or smell in the absence of a negative PCR result; a high temperature; vomiting; and diarrhoea. We restricted our analyses to parents of a child who had exhibited one or more of these stay-at-home symptoms. For parents who had more than one child that met this criteria, we asked them to think about the child that most recently exhibited these symptoms.

We created a single binary variable indicating whether the child had attended at least one activity outside the home (except to get a PCR or LFT COVID-19 test), which included any interaction (inside or outside the home) with someone from another household (“risky behaviour”). Children who had stayed at home (except to get a PCR or LFT COVID-19 test) and had not interacted with someone from another household were categorised as not engaging in risky behaviour.

For all variables, we coded the responses “in another way,” “prefer not to say/answer,” and “don’t know” as missing data.

We ran separate binary logistic regressions to test univariable associations between children engaging in a risky behaviour and: parents’ demographic variables, attitudes about their child and perceptions about illness (as continuous variables). We ran a

For peer review only - http://bmjopen.bmj.com/site/about/guidelines.xhtml

second set of binary logistic regressions adjusting for parent gender, age, region, income, employment status, and education level.

Sensitivity analysis

We re-ran analyses investigating associations with school attendance only (excluding all other out-of-home activities). These analyses were to identify differences in associations between parents of children who engaged in any risky behaviour compared to children who only went to school.

RESULTS

Parents (n=941) reported on symptom(s) for 1,533 children aged four to 17 years. 48% of parents (n=454, 95% CI 45% to 51%) reported that at least one of their children had experienced at least one symptom and 27% (n=251, 95% CI 24% to 30%) reported that at least one child had experienced at least one stay-at-home symptom. Of the 251 children who had at least one stay-at-home symptom, 33% (n=84, 95% CI 28% to 39%) had engaged in risky behaviour.

Table 1 presents the association between our predictor variables and whether children engaged in risky behaviour. Risky behaviour was associated with parent age, with the odds of a child engaging in risky behaviour being 2.9 times higher if the parent was aged 18-35, and 2.4 times higher if the parent was aged 36-45 compared to parents aged 46 or over.

Risky behaviour was associated with parents' agreement with the following statements: their children made their own decisions about when to go to school; children should go to school if they take medication; and if children have a mild symptom of illness, they should go to school.

Sensitivity analysis

When re-running the analyses to look at associations with school attendance only, 227 parents were included. We found school attendance was not associated with parent age, but was associated with parents agreeing that their child did not want to take time off school. No other differences in associations were found between school attendance and risky behaviour and our predictor variables.

For peer review only

Table 1: Associations between parent demographics and attitudes, and whether their child (four to 17 years, with at least one stay-at-home symptom) engaged in risky behaviour (n=251).

Parent demographics and attitudes about sending children to school whilst symptomatic	Level	Children did not engage in risky behaviour (%)	Children engaged in risky behaviour (%)	Odds ratio (95% CI)	P-value	Adjusted odds ratio (95% CI) †	P-Value
Parent gender	Male	73 (64)	41 (36)	1.23 (0.73 to 2.08)	0.44	1.36 (0.76 to 2.44)	0.30
	Female	94 (69)	43 (31)	Reference		Reference	
Parent age	18-35 years	53 (62)	33 (38)	**2.49* (1.18 to 5.26)**	0.02	**2.90* (1.30 to 6.44)**	0.01
	36-45 years	62 (62)	38 (38)	**2.45* (1.18 to 5.09)**	0.02	**2.43* (1.12 to 5.28)**	0.03
	46 years ≥	52 (80)	13 (20)	Reference		Reference	
Parent living region	North of England	46 (70)	20 (30)	0.80 (0.39 to 1.63)	0.54	1.04 (0.49 to 2.22)	0.92
	Midlands England	42 (65)	23 (35)	1.01 (0.50 to 2.03)	0.98	1.03 (0.49 to 2.19)	0.93
	Wales, Scotland, Northern Ireland	31 (76)	15 (33)	0.89 (0.41 to 1.95)	0.78	1.15 (0.50 to 2.63)	0.75
	South of England	48 (65)	26 (35)	Reference		Reference	
Parent household income	≤ £34,999	68 (67)	34 (33)	0.95 (0.55 to 1.63)	0.84	1.04 (0.55 to 1.95)	0.91
	£35,000 ≥	87 (65)	46 (35)	Reference		Reference	
Parent employment status §	Working	131 (66)	69 (35)	1.26 (0.65 to 2.47)	0.49	1.25 (0.56 to 2.77)	0.59
	Not working	36 (71)	15 (29)	Reference		Reference	
Parent education level	≤ A-level	88 (69)	40 (31)	0.82 (0.48 to 1.38)	0.45	0.92 (0.51 to 1.64)	0.77
	Degree ≥	79 (64)	44 (36)	Reference		Reference	
My child has missed too much school since September this year ‡	5-point Likert-scale (1=strongly agree, 5=strongly disagree)	N=166, M=3.11, SD=1.48	N= 83, M=3.17, SD=1.54	1.02 (0.86 to 1.22)	0.79	1.03 (0.85 to 1.26)	0.74
My child is behind at school ‡	5-point Likert-scale (1=strongly agree, 5=strongly disagree)	N=167, M=3.65, SD=1.35	N=84, M=3.21, SD=1.57	**0.81* (0.67 to 0.97)**	0.02	0.84 (0.69 to 1.02)	0.08
My child often says they have symptoms of illnesses when they do not ‡	5-point Likert-scale (1=strongly agree, 5=strongly disagree)	N= 167, M=3.89, SD=1.25	N=84, M=3.54, SD=1.49	**0.83* (0.68 to 0.99)**	0.05	0.84 (0.67 to 1.05)	0.12
Often no-one is available to look after my child if they cannot go to school ‡	5-point Likert-scale (1=strongly agree, 5=strongly disagree)	N=166, M=3.46, SD=1.44	N= 84, M=3.11, SD=1.55	0.85 (0.71 to 1.02)	0.07	0.88 (0.72 to 1.07)	0.20
My child does not want to take time off school ‡	5-point Likert-scale (1=strongly agree, 5=strongly disagree)	N= 162, M=2.43, SD=1.17	N=83, M=2.11, SD=1.14	**0.78* (0.61 to 0.99)**	0.04	0.82 (0.63 to 1.05)	0.12

For peer review only - http://bmjopen.bmj.com/site/about/guidelines.xhtml

My child makes their own decisions about when they go to school ‡	5-point Likert-scale (1=strongly agree, 5=strongly disagree)	N=166, M=4.25, SD=0.98	N=83, M=3.60, SD=1.50	**0.65** (0.52 to 0.81)**	<0.001	**0.65** (0.50 to 0.83)**	<0.001
My child should go to school if they have taken medication (e.g., Calpol, paracetamol) ‡	5-point Likert-scale (1=strongly agree, 5=strongly disagree)	N=164, M=3.04, SD=1.30	N=81, M=2.25, SD=1.20	**0.60** (0.48 to 0.76)**	<0.001	**0.57** (0.44 to 0.75)**	<0.001
If children have common illnesses (e.g., a cold), they should go to school ‡	5-point Likert-scale (1=strongly agree, 5=strongly disagree)	N=166, M=2.30, SD=1.09	N=83, M=2.13, SD=1.03	0.86 (0.66 to 1.11)	0.24	0.83 (0.63 to 1.09)	0.19
Children build up their immunity by mixing with children who have common illnesses (e.g., a cold) ‡	5-point Likert-scale (1=strongly agree, 5=strongly disagree)	N=164, M=1.93, SD=0.88	N=84, M=1.90, SD=1.03	0.97 (0.73 to 1.29)	0.86	0.92 (0.67 to 1.26)	0.61
Other children with common illnesses (e.g., a cold) go to school ‡	5-point Likert-scale (1=strongly agree, 5=strongly disagree)	N=162, M=1.88, SD=0.79	N=83, M=1.84, SD=0.99	0.96 (0.70 to 1.30)	0.77	0.91 (0.65 to 1.27)	0.58
If children have mild symptoms of an illness, they should go to school ‡	5-point Likert-scale (1=strongly agree, 5=strongly disagree)	N=162, M=1.88, SD=0.79	N=80, M=2.46, SD=1.00	**0.77* (0.59 to 0.99)**	0.04	**0.75* (0.56 to 0.99)**	0.05
Going to school is important for my child's mental health ‡	5-point Likert-scale (1=strongly agree, 5=strongly disagree)	N=164, M=1.45, SD=0.67	N=83, M=1.66, SD=0.93	**1.41* (1.01 to 1.97)**	0.04	1.43 (0.99 to 2.06)	0.06
When my child says they are too ill to attend school, I let them stay at home ‡	5-point Likert-scale (1=strongly agree, 5=strongly disagree)	N=161, M=2.58, SD=0.67	N=84, M=2.56, SD=1.15	0.98 (0.77 to 1.25)	0.87	0.96 (0.74 to 1.25)	0.78

*P≤ 0.05 and formatted bold
**P≤ 0.001 and formatted bold.
† Odds ratio adjusted by parent gender, age, living region, household income, employment status, and education level
‡ The exact wording used in the survey
§Working includes students and volunteers.

For peer review only - http://bmjopen.bmj.com/site/about/guidelines.xhtml

DISCUSSION

We found that 33% of children engaged in risky behaviour such as attending school or clubs or socialising with others when experiencing symptoms where it is advised to stay at home. These findings align with previous reports suggesting that school presenteeism is common [9,10]. We also found that risky behaviour was more likely for children with younger parents and for specific parental attitudes about symptoms of illness and their children's behaviour. This is concerning because children who attend school and socialise whilst unwell risk spreading their illness to others, increasing the likelihood that other children will need to take time off school and miss out on extra-curricular and social activities, and putting vulnerable people that they come into contact with at risk.

We found that children were significantly more likely to engage in risky behaviours when their parents believed that children should go to school when their symptoms were mild. This finding mirrors Webster, et al. [11], who found people were more likely to attend school or work with an infectious illness when they perceived their symptoms to be mild. We also found that children were more likely to engage in risky behaviours if a parent felt that attendance at school was appropriate if analgesic medication had been taken. These findings suggest that a key consideration is whether the child will cope with school, rather than whether they might spread illness to others. These findings align with previous research that suggested families were less likely to self-isolate with symptoms that may indicate COVID-19 when parents, a) perceived the symptoms to be mild, b) were unsure of the cause of symptoms and c) perceived a reduced severity of symptoms after taking analgesic medication [14]. A greater focus on the importance of decreasing social mixing when symptomatic may help reduce the incidence and spread of infectious disease outbreaks in schools and to others in the community.

Children were significantly more likely to engage in risky behaviour where their parent reported that the child was responsible for making their own decisions about when they go to school. Educating children, particularly teenagers who may have a greater say over their attendance, about the need to remain at home when ill may be an important part of any future strategy to reduce presenteeism. But this finding may need to be taken with caution; the data were collected in the autumn term when schools re-opened following national school closures due to the pandemic. Reports show that children were keen to get back to school at this time, which may have impacted this result [15]. Still, educating parents on these issues is also important. We found that parents who were aged 45 years or younger were significantly more likely to have a child that engaged in risky behaviour. We can presume that these parents have younger children, and therefore we expect parents to have more control over their child's activities compared to older children. While younger parents may rely on more support from outside the household with childcare, in contrast to previous findings [11]. We did not find a significant association between children who engaged in risky behaviour and whether parents had someone available to look after their child if they could not go to school [11].

For peer review only - http://bmjopen.bmj.com/site/about/guidelines.xhtml

Webster, et al. [11] also found that people who had missed too much work and were concerned about their workload were more likely to attend work with an infectious illness. We were surprised that we did not find a significant association between children who engaged in risky behaviour and parents who felt their child had missed too much school and were behind in school. This was also replicated in our sensitivity analyses, that focused on children who had attended school and excluded all other types of risky behaviour. We cannot be sure why our results contrast with previous research, but this could be due to the differences between parents not attending work (e.g., financial implications) and children not attending school (e.g., educational impacts).

There were several limitations to this study. First, the data were drawn from a non-probability sample. Whether the sample was behaviourally and psychologically representative of the wider population of parents in the UK is unknown [16], although we have no reason to suspect that the associations within the data cannot be generalised [17]. Second, our participants were mainly of white ethnicity (91%, n=224/246, n=5 were missing data) and we were unable to include ethnicity in our analysis due to small case numbers. Third, we did not include child demographics in our analysis due to survey space limitations. Fourth, our data collection occurred during a pandemic, with a substantial focus on the importance of remaining home when potentially infectious and after an extended period of home-schooling. How rates and predictors of presenteeism will change as we emerge from the pandemic is unknown.

In conclusion, one-third of children with symptoms that indicated an infectious illness engaged in activities outside the home, including going to school or socialising with others. This behaviour goes against UK Government advice that is in place to prevent the spread of disease. We found that younger parents, children who made their own decisions about school attendance, children who had taken analgesic medication and having mild symptoms were risk factors for engaging in risky behaviour. Parents and teenagers need guidance to help them make informed decisions about school attendance whilst unwell to reduce school presenteeism.

Data availability statement

The data set and survey questions related to this study can be accessed via the King's College London research data repository, KORDS (DOI 10.18742/19519444). The larger five-wave dataset will be archived with the UK Data Service after the end of the project, reference ES/V015494/1, in May 2022. This dataset can be matched with the full dataset on the ID variable.

Author contributions

All authors contributed to the conceptualisation of the study and approved the final draft. LW designed the survey, analysed the data and drafted the manuscript. LES, RA and GJR designed the survey and edited the manuscript. RKW edited the manuscript. RKW, RA and GJR supervised LW.

Funding statement

For peer review only - http://bmjopen.bmj.com/site/about/guidelines.xhtml

This study was funded by the Economic and Social Research Council [grant number ES/P000703/1] and by the National Institute for Health and Care Research Health Protection Research Unit (NIHR HPRU) in Emergency Preparedness and Response [grant number NIHR200890], a partnership between the UK Health Security Agency, King's College London and the University of East Anglia. The views expressed are those of the author(s) and not necessarily those of the NIHR, UKHSA or the Department of Health and Social Care. For the purpose of open access, the author has applied a Creative Commons Attribution (CC BY) licence to any Author Accepted Manuscript version arising.

The funders had no role in study design, data collection, data analysis, data interpretation, or writing of the manuscript. The corresponding author had full access to all the data and had final responsibility for the decision to submit for publication.

Conflict of interest disclosure

GJR, RA and LS participate in the UK's Scientific Advisory Group for Emergencies, or its subgroups. These groups did not fund the study or authors. RA is an employee of the UK Health Security Agency.

REFERENCES

1. National Health Service. Is my child too ill for school? 2021 [updated 1 April. Available from: https://www.nhs.uk/live-well/healthy-body/is-my-child-too-ill-for-school/ accessed 3 February 2022.
2. HM Government. Health protection in schools and other childcare facilities 2021 [updated 3 March 2021. Available from: https://www.gov.uk/government/publications/health-protection-in-schools-and-other-childcare-facilities accessed 9 February 2022.
3. HM Government. Stay at home: guidance for households with possible or confirmed coronavirus (COVID-19) infection 2022 [updated 1 February. Available from: https://www.gov.uk/government/publications/covid-19-stay-at-home-guidance/stay-at-home-guidance-for-households-with-possible-coronavirus-covid-19-infection accessed 3 February 2022.
4. HM Government. Prime Minister's statement on coronavirus (COVID-19): 23 March 2020: HM Government,; 2020 [Available from: https://www.gov.uk/government/speeches/pm-address-to-the-nation-on-coronavirus-23-march-2020 accessed 11 June 2020.
5. HM Government. Norovirus outbreaks increasing in England, 2022.
6. Cleary V, Slaughter R, Heathcock R. An Infection Control Programme in Primary Schools and the Wider Public Health Impact. *Journal of Infection Prevention* 2003;4(5):11-17. doi: 10.1177/175717740300400503
7. Thomson RM, Henderson HJ, Smith-Palmer A. An outbreak of Salmonella Saintpaul in a Scottish childcare facility: the influence of parental under-reporting. *BMC Infectious Diseases* 2019;19(1):847. doi: https://dx.doi.org/10.1186/s12879-019-4516-z
8. Joseph C, Noah N, White J, et al. A review of outbreaks of infectious disease in schools in England and Wales 1979–88. *Epidemiology and Infection* 1990;105(2):419-34. doi: 10.1017/s0950268800048007

For peer review only - http://bmjopen.bmj.com/site/about/guidelines.xhtml

9. Johansen V. Sick and still at school: an empirical study of sickness presence among students in Norwegian secondary school. *BMJ Open* 2015;5(9):e008290. doi: https://dx.doi.org/10.1136/bmjopen-2015-008290
10. BUPA. Bupa report reveals that half of all UK parents send children to school when ill 2010 [updated 26 October. Available from: https://pressreleases.responsesource.com/news/60199/bupa-report-reveals-that-half-of-all-uk-parents-send/ accessed 11 June 2020.
11. Webster RK, Liu R, Karimullina K, et al. A systematic review of infectious illness Presenteeism: prevalence, reasons and risk factors. *BMC Public Health* 2019;19(1):799. doi: 10.1186/s12889-019-7138-x
12. Ipsos MORI. About us n.d [Available from: https://www.ipsos.com/en-uk/about-us accessed 17 February 2022.
13. von Elm E, Altman DG, Egger M, et al. The Strengthening the Reporting of Observational Studies in Epidemiology (STROBE) Statement: Guidelines for Reporting Observational Studies. *Annals of Internal Medicine* 2007;147(8):573-77. doi: 10.7326/0003-4819-147-8-200710160-00010
14. Woodland L, Mowbray F, Smith LE, et al. What influences whether parents recognise COVID-19 symptoms, request a test and self-isolate: A qualitative study. *PLOS ONE* 2022;17(2):e0263537. doi: 10.1371/journal.pone.0263537
15. Children's Commissioner. Some sort of normal 2020 [updated 2 November. Available from: https://www.childrenscommissioner.gov.uk/education/ accessed 11 March 2022.
16. Epstein WM. Response bias in opinion polls and American social welfare. *The Social Science Journal* 2006;43(1):99-110. doi: 10.1016/j.soscij.2005.12.010
17. Kohler U. Possible Uses of Nonprobability Sampling for the Social Sciences. *Survey Methods: Insights from the Field* 2019 [updated 2 April. Available from: https://surveyinsights.org/?p=10981 accessed 29 July 2020.

For peer review only

Text S1: Survey questions

Standard demographic variables:

1. What is your date of birth?

1. YEAR
2. 1910 1910
3. ...
4. 2015 2015
5. MONTH
6. January
7. February
8. March
9. April
10. May
11. June
12. July
13. August
14. September
15. October
16. November
17. December

2. Which of the following describes how you think of yourself?

1. Male
2. Female
3. In another way
4. Prefer not to answer

3. Where do you live? Please note: This question may be considered personal. We would like to remind you that your participation is strictly voluntary and that your responses are used for research purposes only. The answers that you provide will be presented in aggregate form and none of them will be linked back to you in any way. All data will be collected and processed in adherence to the Market Research Society's Code of Conduct and the General Data Protection Regulation (GDPR).

1. Postcode
2. Postal Town
3. Prefer Not to Answer

4. Where do you live?

1. North East
2. North West
3. Yorkshire and The Humber
4. West Midlands
5. East Midlands
6. East of England

For peer review only

For peer review only - http://bmjopen.bmj.com/site/about/guidelines.xhtml

7. South West
8. South East
9. Greater London
10. Wales
11. Scotland
12. Northern Ireland

5. How many people are living or staying at your current address? (Include yourself and any other adults or children who are currently living or staying at this address for at least two months)

1. 1
2. 2
3. 3
4. 4
5. 5
6. 6
7. 7
8. 8
9. 9
10. 10
11. 11
12. 12+

6. How many children under the age of 18 are living in your household? Please reference only the children for which you are the parent or legal guardian. (If there are no children under 18 in your household, please type 0)

7. Please provide us with the following information about the children under the age of 18 in your household. Please reference only the children for which you are the parent or legal guardian.

1. Relationship
2. Parent (biological or adopted)
3. Legal guardian
4. Other (e.g. step-child)
5. Gender
6. Boy
7. Girl
8. Year of birth
9. Month of birth

8. What is your highest level of education attained?

1. Primary school
2. Secondary school (age under 15 years old)
3. GNVQ / GSVQ / GCSE/ SCE standard.
4. NVQ1, NVQ2
5. NVQ3/ SCE Higher Grade/ Advanced GNVQ/ GCE A/AS or similar.
6. NVQ4 / HNC / HND / Bachelor's degree or similar.
7. NVQ5 or post-graduate diploma.

For peer review only

9. The next question may be considered personal, but it is not mandatory to answer. If you do, we assure you that your responses will be kept strictly confidential and used for research purposes only. What is the COMBINED TOTAL ANNUAL INCOME (pre-tax) earned by all members of your household? Please include all your income sources : salaries, scholarships, pension and Social Security benefits, dividends from shares, income from rental properties, child support and alimony etc.

1. Under £5,000
2. £5,000 - £9,999
3. £10,000 - £14,999
4. £15,000 - £19,999
5. £20,000 - £24,999
6. £25,000 - £34,999
7. £35,000 - £44,999
8. £45,000 - £54,999
9. £55,000 - £99,999
10. £100,000 or more
11. Prefer not to answer

10. What is your current employment status?

1. Employed full-time
2. Employed part-time
3. Self employed
4. Unemployed but looking for a job
5. Unemployed and not looking for a job/Long-term sick or disabled
6. Full-time parent, homemaker
7. Retired
8. Student/Pupil

11. In which of the below categories does your occupation fall? If retired or unemployed, please indicate the category closest to your previous occupation.

Responses copied from https://www.ilo.org/public/english/bureau/stat/isco/isco88/

12. What is the occupation of the person with the highest income? If retired or unemployed, please indicate the category closest to his/her previous occupation.

Responses copied from https://www.ilo.org/public/english/bureau/stat/isco/isco88/

In this survey, some questions will be about health and perceptions about, coronavirus, politics, religious beliefs, etc. A "Prefer not to answer" option will be available for you to select, if the case. Ipsos is running the survey and collecting such data in order to understand attitudes towards public health measures in the context of the coronavirus pandemic, and trust in their efficacy.

Ipsos has been commissioned by the University of Bristol and King's College London to carry out this research. Information collected through the survey will be made available to the team of researchers at the University of Bristol and King's College London in an anonymised format, unless you provide your permission to pass personal data to them. At

For peer review only - http://bmjopen.bmj.com/site/about/guidelines.xhtml

no point will any information that would allow you to be identified be made available to the public.

Participation is completely voluntary and you may withdraw your consent at any time. Your survey answers will be combined with the answers from all other participants and used for social research purposes only, and your personal data will be held for no longer than 12 months within Ipsos and no longer than April 2023 from researchers at the University of Bristol and King's College London.

Do you accept the collection of personal information and of health and perceptions about coronavirus related data?

1. *Yes, I accept*
2. *No, I do not accept*

13. Which of these applies to your home?

1. Being bought on a mortgage
2. Owned outright by household
3. Rented from a local authority
4. Rented from a housing association/trust
5. Rented from a private landlord
6. Other
7. Refused/don't know

The next question may be considered personal, but it is not mandatory to answer. If you do, we assure you that your responses will be kept strictly confidential and used for research purposes only.

14. What is your ethnic group?

Please select only one

White

1. English / Welsh / Scottish / Northern Irish / British
2. Irish
3. Gypsy or Irish Traveller
4. Any other White background

Mixed / multiple ethnic groups

5. White and Black Caribbean
6. White and Black African
7. White and Asian
8. Any other Mixed / multiple ethnic background

Asian / Asian British

9. Indian
10. Pakistani

For peer review only

For peer review only - http://bmjopen.bmj.com/site/about/guidelines.xhtml

11. Bangladeshi
12. Chinese
13. Any other Asian background

Black / African / Caribbean / Black British

14. African
15. Caribbean
16. Any other Black / African / Caribbean background

Other ethnic group

17. Arab
18. Any other ethnic group, please write in
19. Prefer not to answer

For peer review only

Survey questions

For the next set of questions, we would like you to think about your [number of children] youngest children who usually attend school.

15. Since *about* [Scotland only] the start of the school year (September 2021), please tick, for each child, any new symptoms that they developed for any reason (e.g. a cold, allergic reaction, coronavirus, etc.), even if some of the symptoms were mild. If they have developed new symptoms more than once during that period (for example if they have had a cold in one week, and an allergic reaction a couple of weeks later), please just tell us about the most severe set of symptoms that they had (for example, tell us about the cold, or the allergic reaction, whichever seemed worse). Please tick any that apply.

1. New, continuous cough
2. High temperature / fever
3. Diarrhoea
4. Nausea / feeling sick
5. Vomiting / being sick
6. Earache
7. A rash
8. Loss of sense of smell (fully or partial)
9. Loss of taste
10. Sore throat
11. Headache
12. Muscle ache
13. Breathlessness, tight chest, or wheezing
14. Feeling tired or exhausted
15. I don’t have any (more) children who usually attend school
16. None of these
17. Don’t know
18. Prefer not to say

For peer review only - http://bmjopen.bmj.com/site/about/guidelines.xhtml

1. Child 1 (youngest)
2. Child 2
3. Child 3
4. Child (oldest)

16. Please think about your child who most recently developed symptoms. When they had symptoms, did they do any of the following?
Please tick any that apply. Do not count things that they did online.

1. Went to school
2. Went to a club or lesson outside of school
3. Visited someone from another household
4. Someone from another household visited the child
5. Someone from another household visited our household
6. Took a lateral flow test (LFT) to see whether they had coronavirus (this is a rapid test that gives results in 30 minutes)
7. Took a polymerase chain reaction (PCR) test to see whether they had coronavirus (this is a test that is sent off to a laboratory)
8. Took both a lateral flow test (LFT, a rapid test that gives results in 30 minutes) and a polymerase chain reaction (PCR) test (a test that you send off to the laboratory) to see whether they had coronavirus
9. Took a test to see whether they had coronavirus, but I am not sure what type of test it was
10. None of these
11. Prefer not to say

17. Please still think about your child who most recently developed symptoms. You said your child had taken at least one rapid lateral flow test (LFT). Were any of the results positive?

1. Yes, this child had one or more rapid lateral flow tests (LFT) and had a positive result
2. None of the rapid lateral flow tests (LFT) for this child had a positive result
3. Don't know
4. Prefer not to say

18. Please still think about your child who most recently developed symptoms. You said your child had taken a polymerase chain reaction (PCR) test to see whether they had coronavirus. What was the result?

1. My child tested positive
2. My child tested negative
3. The result was inconclusive
4. I have not received the results
5. Don't know
6. Prefer not to say

For peer review only - http://bmjopen.bmj.com/site/about/guidelines.xhtml

19. Thinking about the same child who most recently developed symptoms, to what extent, if at all, do you agree or disagree with the following statements about this child, as a result of having these symptoms?

1. My child has missed too much school since September this year
2. My child is behind at school
3. My child often says they have symptoms of illnesses when they do not
4. Often no-one is available to look after my child if they cannot go to school
5. My child does not want to take time off school
6. My child makes their own decisions about when they go to school
7. My child should go to school if they have taken medication (e.g., Calpol, paracetamol)

    1. Strongly agree
    2. Tend to agree
    3. Neither agree nor disagree
    4. Tend to disagree
    5. Strongly disagree
    6. Don't know
    7. Prefer not to say

20. Now thinking in general, to what extent, if at all, do you agree or disagree with the following statements?

1. If children have common illnesses (e.g., a cold), they should go to school
2. Children build up their immunity by mixing with children who have common illnesses (e.g., a cold)
3. Other children with common illnesses (e.g. a cold) go to school
4. If children have mild symptoms of an illness, they should go to school
5. Going to school is important for my child's mental health
6. When my child says they are too ill to attend school, I let them stay at home

    1. Strongly agree
    2. Tend to agree
    3. Neither agree nor disagree
    4. Tend to disagree
    5. Strongly disagree
    6. Don't know
    7. Prefer not to say

For peer review only - http://bmjopen.bmj.com/site/about/guidelines.xhtml

Reporting checklist for cross sectional study.

Based on the STROBE cross sectional guidelines.

Instructions to authors

Complete this checklist by entering the page numbers from your manuscript where readers will find each of the items listed below.

Your article may not currently address all the items on the checklist. Please modify your text to include the missing information. If you are certain that an item does not apply, please write "n/a" and provide a short explanation.

Upload your completed checklist as an extra file when you submit to a journal.

In your methods section, say that you used the STROBE cross sectionalreporting guidelines, and cite them as:

von Elm E, Altman DG, Egger M, Pocock SJ, Gotzsche PC, Vandenbroucke JP. The Strengthening the Reporting of Observational Studies in Epidemiology (STROBE) Statement: guidelines for reporting observational studies.

		Reporting Item	Page Number
**Title and abstract**			
Title	#1a	Indicate the study's design with a commonly used term in the title or the abstract	1
Abstract	#1b	Provide in the abstract an informative and balanced summary of what was done and what was found	2
**Introduction**			
Background / rationale	#2	Explain the scientific background and rationale for the investigation being reported	3
Objectives	#3	State specific objectives, including any prespecified hypotheses	3
**Methods**			
Study design	#4	Present key elements of study design early in the paper	3
Setting	#5	Describe the setting, locations, and relevant dates, including periods of	4

For peer review only

		recruitment, exposure, follow-up, and data collection	
Eligibility criteria	#6a	Give the eligibility criteria, and the sources and methods of selection of participants.	3
	#7	Clearly define all outcomes, exposures, predictors, potential confounders, and effect modifiers. Give diagnostic criteria, if applicable	4,5
Data sources / measurement	#8	For each variable of interest give sources of data and details of methods of assessment (measurement). Describe comparability of assessment methods if there is more than one group. Give information separately for for exposed and unexposed groups if applicable.	5
Bias	#9	Describe any efforts to address potential sources of bias	5
Study size	#10	Explain how the study size was arrived at	5
Quantitative variables	#11	Explain how quantitative variables were handled in the analyses. If applicable, describe which groupings were chosen, and why	5
Statistical methods	#12a	Describe all statistical methods, including those used to control for confounding	5
Statistical methods	#12b	Describe any methods used to examine subgroups and interactions	5
Statistical methods	#12c	Explain how missing data were addressed	5
Statistical methods	#12d	If applicable, describe analytical methods taking account of sampling strategy	5
Statistical methods	#12e	Describe any sensitivity analyses	6
**Results**			
Participants	#13a	Report numbers of individuals at each stage of study—eg numbers potentially eligible, examined for eligibility, confirmed eligible, included in the study, completing follow-up, and analysed. Give information separately for for exposed and unexposed groups if applicable.	6
Participants	#13b	Give reasons for non-participation at each stage	6
Participants	#13c	Consider use of a flow diagram	n/a

For peer review only

Descriptive data	#14a	Give characteristics of study participants (eg demographic, clinical, social) and information on exposures and potential confounders. Give information separately for exposed and unexposed groups if applicable.	6
Descriptive data	#14b	Indicate number of participants with missing data for each variable of interest	7,8
Outcome data	#15	Report numbers of outcome events or summary measures. Give information separately for exposed and unexposed groups if applicable.	7,8
Main results	#16a	Give unadjusted estimates and, if applicable, confounder-adjusted estimates and their precision (eg, 95% confidence interval). Make clear which confounders were adjusted for and why they were included	7,8
Main results	#16b	Report category boundaries when continuous variables were categorized	7,8
Main results	#16c	If relevant, consider translating estimates of relative risk into absolute risk for a meaningful time period	n/a
Other analyses	#17	Report other analyses done—e.g., analyses of subgroups and interactions, and sensitivity analyses	6
**Discussion**			
Key results	#18	Summarise key results with reference to study objectives	9
Limitations	#19	Discuss limitations of the study, taking into account sources of potential bias or imprecision. Discuss both direction and magnitude of any potential bias.	10
Interpretation	#20	Give a cautious overall interpretation considering objectives, limitations, multiplicity of analyses, results from similar studies, and other relevant evidence.	9
Generalisability	#21	Discuss the generalisability (external validity) of the study results	10
**Other Information**			
Funding	#22	Give the source of funding and the role of the funders for the present study and, if applicable, for the original study on which the present article is based	11

The STROBE checklist is distributed under the terms of the Creative Commons Attribution License CC-BY.
This checklist was completed on 03. January 2023 using https://www.goodreports.org/, a tool made by the EQUATOR Network in collaboration with Penelope.ai

For peer review only

BMJ Open

Why do children attend school, other activities or socialise when they have symptoms of an infectious illness? A cross-sectional survey

Journal:	*BMJ Open*
Manuscript ID	bmjopen-2023-071599.R1
Article Type:	Original research
Date Submitted by the Author:	26-Jul-2023
Complete List of Authors:	Woodland, Lisa; King's College London, Psychological Medicine<br>Smith, Louise; King's College London, Psychological Medicine<br>Webster, Rebecca; The University of Sheffield, Psychology<br>Amlôt, Richard; UK Health Security Agency<br>Rubin, G James; King's College London, Psychological Medicine
**Primary Subject Heading**:	Health policy
Secondary Subject Heading:	Infectious diseases
Keywords:	INFECTIOUS DISEASES, COVID-19, Health policy < HEALTH SERVICES ADMINISTRATION & MANAGEMENT

SCHOLARONE™ Manuscripts

I, the Submitting Author has the right to grant and does grant on behalf of all authors of the Work (as defined in the below author licence), an exclusive licence and/or a non-exclusive licence for contributions from authors who are: i) UK Crown employees; ii) where BMJ has agreed a CC-BY licence shall apply, and/or iii) in accordance with the terms applicable for US Federal Government officers or employees acting as part of their official duties; on a worldwide, perpetual, irrevocable, royalty-free basis to BMJ Publishing Group Ltd ("BMJ") its licensees and where the relevant Journal is co-owned by BMJ to the co-owners of the Journal, to publish the Work in this journal and any other BMJ products and to exploit all rights, as set out in our licence.

The Submitting Author accepts and understands that any supply made under these terms is made by BMJ to the Submitting Author unless you are acting as an employee on behalf of your employer or a postgraduate student of an affiliated institution which is paying any applicable article publishing charge ("APC") for Open Access articles. Where the Submitting Author wishes to make the Work available on an Open Access basis (and intends to pay the relevant APC), the terms of reuse of such Open Access shall be governed by a Creative Commons licence – details of these licences and which Creative Commons licence will apply to this Work are set out in our licence referred to above.

Other than as permitted in any relevant BMJ Author's Self Archiving Policies, I confirm this Work has not been accepted for publication elsewhere, is not being considered for publication elsewhere and does not duplicate material already published. I confirm all authors consent to publication of this Work and authorise the granting of this licence.

For peer review only

Why do children attend school, other activities or socialise when they have symptoms of an infectious illness? A cross-sectional survey

For peer review only

Lisa Woodland MSc,[1,4*] Weston Education Centre, Cutcombe Road, London SE5 9RJ, UK (PhD Researcher in Psychological Medicine)

Louise E Smith PhD,[1,4] Weston Education Centre, Cutcombe Road, London SE5 9RJ, UK (Research Associate in Psychological Medicine)

Rebecca K Webster PhD,[2] Cathedral Court, 1 Vicar Lane, Sheffield, S1 2LT, UK (Lecturer, Department of Psychology)

Richard Amlôt PhD,[3,4] Behavioural Science and Insights Unit (BSIU), Porton Down, Salisbury, SP4 0JG, UK (Deputy Director, Behavioural Science and Insights Unit (BSIU))

G James Rubin PhD,[1,4] Weston Education Centre, Cutcombe Road, London SE5 9RJ, UK (Professor of Psychology & Emerging Health Risks)

[1] King's College London

[2] University of Sheffield

[3] UK Health Security Agency

[4] NIHR Health Protection Research Unit in Emergency Preparedness and Response

Correspondence to: Lisa Woodland, King's College London, Weston Education Centre, Cutcombe Road, London SE5 9RJ, UK, lisa.woodland@kcl.ac.uk

Word Count: 4,443

Key words: Infectious diseases; non-adherence; children; presenteeism; COVID-19; diarrhoea; vomiting

For peer review only - http://bmjopen.bmj.com/site/about/guidelines.xhtml

ABSTRACT

Objectives

To prevent the spread of infectious disease, children are typically asked not to attend school, clubs or other activities, or socialise with others whilst they have specific symptoms. Despite this, many children continue to participate in these activities whilst symptomatic.

Design and setting

We commissioned a national cross-sectional survey with data collected between 19 November and 18 December 2021.

Participants

Eligible parents (n=941) were between 18 and 75 years of age, lived in the UK and had at least one child aged between four and 17 years old. Parents were recruited from a pre-existing pool of potential respondents who had already expressed in interest in receiving market research surveys.

Outcome measures

Parents were asked whether their children had exhibited either recent vomiting, diarrhoea, high temperature/fever, a new continuous cough, a loss or change to their sense of taste or smell in the absence of a negative (PCR) COVID-19 test ("stay-at-home symptoms") since September 2019 and whether they attended school, engaged in other activities outside the home or socialised with members of another household whilst symptomatic ("non-adherent"). We also measured parent's demographics and attitudes about illness.

Results

One-third (33%, n=84/251, 95% CI 28% to 39%) of children were "non-adherent" in that they had attended activities outside the home or socialised when they had stay-at-home symptoms. Children were significantly more likely to be non-adherent when parents: were aged 45 and younger; allowed their children to make their own decisions about school attendance; agreed that their child should go to school if they took over the counter medication; or believed that children should go to school if they have mild symptoms of illness.

Conclusion

To reduce the risk of spreading disease, parents and teenagers need guidance to help them make informed decisions about engaging in activities and socialising with others whilst unwell.

STRENGTHS AND LIMITATIONS OF THIS STUDY

- A strength of the study is that we investigated reported behaviour, rather than intentions.

For peer review only

For peer review only - http://bmjopen.bmj.com/site/about/guidelines.xhtml

- The study used a non-probability sample, thus there is a risk of some sampling bias.
- Data about children's demographics were not collected, which limits our ability to interpret the data.

For peer review only

For peer review only - http://bmjopen.bmj.com/site/about/guidelines.xhtml

INTRODUCTION

In order to reduce the spread of infectious illness within schools, children who have specific symptoms, including fever, diarrhoea and vomiting, are commonly advised to remain at home [1,2]. While this has been the case for many years, this message was made more urgent by the COVID-19 pandemic. In the UK, throughout most of 2020 and 2021, anyone with a new continuous cough, a high temperature, or a loss or change to their sense of taste or smell were asked not to attend school or work nor to interact with people outside their household unless they had a negative COVID-19 polymerase chain reaction (PCR) test result [3,4]. This message is also important for other symptoms of infectious illnesses. For instance, in February 2022, cases of norovirus were 48% higher than expected in educational settings [5], resulting in a warning to the public about the health threat [5]. Children are particularly susceptible to infections because their immune systems are developing [6], they are often in close contact with other children, and although they can adhere to hygiene practices [7], they are at risk of infections [2,8,9]. Preventing ill children from mixing with others is essential in mitigating the spread of infection [10,11]. The UK Government has issued guidance on how long a child should not attend school for when they show symptoms of an infectious illness [2].

A systematic review indicates that a large proportion of symptomatic children may be attending school (known as "school-based presenteeism") [12]. For example, one survey of 3,040 secondary school pupils in Norway reported that 58% had attended school in the past year despite feeling so ill that they should have stayed at home [13]. One in six British parents reported that they would send their child to school even if they were currently experiencing diarrhoea or vomiting [14]. The systematic review identified five themes that were shown to impact school-based presenteeism: children's characteristics, children's and parents' motivations and attitudes towards school; organisational factors and school sickness policy [12]. Another systematic review found similar findings and suggested that people are more likely to attend school or work with symptoms of an infectious illness if they: are unsure of the sickness guidelines; are worried about disciplinary action; are unable to find alternative child care; are concerned about their workload; feel they have missed too much work; perceive a culture of presenteeism in the organisation; and perceive their illness to be mild or non-infectious [15]. The studies included in the systematic reviews focus only on work or school-based presenteeism and the findings in relation to children in the UK are limited. However, hypotheses can be drawn; and children who have positive motivations towards school, worry about school attendance and when the symptoms of illness are mild, will be more at risk of non-adherence.

In this study, we aimed to identify the proportion of children who were non-adherent (e.g., attended school, clubs, or other activities or who socialised with people outside their household) while they had symptoms that should require them to remain at home. We also aimed to identify the possible parent and child-level risk factors for engaging in these activities. The results of which can then be used to inform interventions that target these risk factors as a means to prevent the spread of infectious diseases.

For peer review only - http://bmjopen.bmj.com/site/about/guidelines.xhtml

METHODS

Design

We used data from a longitudinal study about perceptions of COVID-19, trust in public officials and other health attitudes and behaviours. We commissioned Ipsos MORI [16] to conduct this national cross-sectional online survey of 5,000 participants at each wave. This paper is based on data from wave 5, which collected data between 19 November and 18 December 2021.

Participants

Non-probability sampling was used for ease of participant recruitment and to limit study costs. Participants were recruited from a pre-existing pool of potential respondents who had already expressed an interest in receiving market research surveys. Participants were recruited by advertising via platforms such as social media, online gaming sites and news outlets. In Europe, 38% of the volume is recruited through social media, 26% through self-recruitment and referral and 36% through affiliate networks and media agencies. Further information about the company's recruitment process can be found in their guidance [17].

Participants aged between 18 and 75, and who lived in the UK were eligible for the survey. Quotas were set on the main sample: age interlocked by gender; government office region; working status; and social grade based on PAMCo 4 (October 2018 to September 2019) data[18]. One percent of participants were removed due to Ipsos MORI's quality control procedures [17]. The survey company perform automated quality checks, such as country geo-IP validation and removing duplicate email identifications. Further checks were made on the final data set, and data that indicated inconsistencies, such as straight-lining, when the participant selects the same response throughout the survey, and incomplete surveys were removed.

Recruitment followed a complex pattern, in which responders from earlier waves were invited to participate and additional respondents from the market research panel were then invited to take part to take the place of non-responders. Response rates are not provided because they are not an accurate indicator of bias in quota samples: given that the underlying sample frame consists of people who have previously self-selected to receive invitations to take part in surveys, understanding whether the achieved sample (within each quota) is representative of the sample frame provides no information as to whether it is also representative of the wider general population.

Participants were paid between €1 and €1.50 for completing the survey.

The target population was parents of dependent children. As this was a sub-sample of a wider survey the participants and sample size were based on the needs for the main study, which set quotas to obtain a sample broadly representative of the UK adult population. In our parent sample: 80% were employed, in the UK 69% of parents are employed in lone parent households and both parents are employed in 74% of couple households [19]; 54% earned £35,000 and over and the average disposable income for UK households with children was £35,049 [20].

For peer review only - http://bmjopen.bmj.com/site/about/guidelines.xhtml

Study materials

The wording of all survey items is available in the supplementary materials (Text S1).

Participant demographics

We asked parents to report their gender, age, postcode (to derive region), household income, employment status, ethnicity, and level of education. A non-response option was available for each of these demographic questions (“prefer not to say,” “prefer not to answer,” and for gender only, parents could also respond “in another way”). Due to space limitations in the survey, we did not collect child demographic data.

Identification of children with symptoms of illness

Parents were asked to consider their four (or fewer) youngest children aged between four and 17 years and to report for each child any symptoms they had experienced since the start of the school year (September 2021). We restricted the number of children parents could report on to four to allow most parents to report the symptoms for all of their children whilst limiting the length of the survey. In Scotland, the academic year started in August 2021, therefore, participants in Scotland were asked about their child’s symptoms since “*about* the start of the school year (September 2021)”. If a child had experienced multiple bouts of illness during that period, we asked parents to report the one set of symptoms they perceived as most severe. Parents were asked to report all symptoms that applied to their child from a list of 14 symptoms of infectious illnesses and ailments common in children, listed by the UK Government [2].

Children’s activities whilst symptomatic

Parents who had at least one symptomatic child were asked to consider the child who most recently exhibited symptoms and to report whether, when they had symptoms, they engaged in any of the following activities (excluding online): going to school; going to a club or lesson outside of school; visiting someone from another household; having someone from another household visit them; having someone from another household visit the household in general. Parents were asked to “tick any [activity] that applied,” or they could respond “none of these” or “prefer not to say.”

We also asked parents whether their child had taken a COVID-19 test (lateral flow (LFT) test or a PCR test) while symptomatic, and if so, the result of the test.

Parent attitudes about their child and perceptions about illness

We asked parents 13 statements about their attitudes concerning their child (e.g., my child has missed too much school since September this year) and about common perceptions about illness (e.g., other children with common illnesses (e.g., a cold) go to school). We asked parents to respond to each statement on a five-point Likert scale from “strongly agree” to “strongly disagree”; they could also respond “don’t know” or “prefer not to say.”

For peer review only - http://bmjopen.bmj.com/site/about/guidelines.xhtml

Ethics

The research was approved by the Faculty of Social Sciences and Law, University of Bristol (approval code 116976 and 8273). Participants provided informed consent. Data were anonymised and only researchers related to this study had access.

Reporting

We used the STROBE cross sectional checklist when writing this manuscript [21].

Patient and public involvement

None

Analysis

Analysis was conducted in SPSS [22]. We created binary and multinominal variables for parent demographics (presented in Table 1).

We recoded symptoms that necessitated the child to “stay-at-home” according to Government guidance as being: a new, continuous cough or a loss or change to their sense of taste or smell in the absence of a negative PCR result; a high temperature; vomiting; and diarrhoea. We restricted our analyses to parents of a child who had exhibited one or more of these stay-at-home symptoms.

We created a single binary variable indicating whether the child had attended at least one activity outside the home (except to get a PCR or LFT COVID-19 test), which included any interaction (inside or outside the home) with someone from another household (“non-adherence”). Children who had stayed at home (except to get a PCR or LFT COVID-19 test) and had not interacted with someone from another household were categorised as adherent.

For all variables, we coded the responses “in another way,” “prefer not to say/answer,” and “don’t know” as missing data. Participants had to complete each question before moving onto the next; as such there were no other missing data.

We ran separate binary logistic regressions to test univariable associations between children’s adherence and: parents’ demographic variables, attitudes about their child and perceptions about illness (as continuous variables). We ran a second set of binary logistic regressions adjusting for parent gender, age, region, income, employment status, and education level. The potential cofounders were entered into the regression model at the same time for every test.

Sensitivity analysis

We re-ran analyses investigating associations with school attendance only (excluding all other out-of-home activities and social interactions). These analyses were to identify differences in associations between parents of children who were non-adherent in any behaviour compared to children who were non-adherent due to only attending school.

RESULTS

For peer review only - http://bmjopen.bmj.com/site/about/guidelines.xhtml

4,962 participants completed the main survey, of whom 941 (19%) indicated that they had a child aged between four and 17 years. This proportion of respondents is as expected as 22% of households in the UK have a dependent child [23].

Of these 941 parents, 251 reported that their child had experienced symptom(s) that required them to stay at home.

Parents (n=941) reported on symptom(s) for 1,533 children aged four to 17 years. 48% of parents (n=454, 95% CI 45% to 51%) reported that at least one of their children had experienced at least one symptom and 27% (n=251, 95% CI 24% to 30%) reported that at least one child had experienced at least one stay-at-home symptom. Of the 251 children who had at least one stay-at-home symptom, 33% (n=84, 95% CI 28% to 39%) were non-adherent.

Most parents included in this study were female (55%, n = 137), aged between 36 and 45 years (40%, n = 100) and earned over £35,000 (57%, n = 133) (males: 45%, n = 114; aged 18 to 35 years: 34%, n = 86, aged over 46 years: 26%, n = 65; earned under £34,999: 43%, n = 102). Table 1 presents associations between predictor variables and whether children went to school, engaged in other activities, or socialised with non-household members (non-adherent). Non-adherence was moderately associated with younger parent age, with the odds of a child being non-adherent 2.9 times higher if the parent was aged 18-35, and 2.4 times higher if the parent was aged 36-45 compared to parents aged 46 or over.

Non-adherence was associated with parents' agreement with the following statements: their children made their own decisions about when to go to school; children should go to school if they take medication; and if children have a mild symptom of illness, they should go to school. Table 1 reports the exact statement wording, with lower values reflecting agreement with that statement.

Table 2 shows the frequencies of children's adherence and non-adherence for each of the behaviours that were then grouped into our outcome variable.

Sensitivity analysis

When re-running the analyses to look at associations with school attendance only, 227 parents were included. Fewer participants were included in the sensitivity analysis because not all 251 children attended school while symptomatic with stay-at-home symptoms. We found school attendance was not associated with parent age, but was associated with parents agreeing that their child did not want to take time off school. No other differences in associations were found between school attendance and non-adherence and our predictor variables.

For peer review only - http://bmjopen.bmj.com/site/about/guidelines.xhtml

Table 1: Associations between parent demographics and attitudes, and whether their child (four to 17 years, with at least one stay-at-home symptom) were non-adherent (n=251).

Parent demographics and attitudes about sending children to school whilst symptomatic	Level	Children were adherent (%)	Children were non-adherent (%)	Odds ratio (95% CI)	P-value	Adjusted odds ratio (95% CI) †	P-Value
Parent gender	Male	73 (64)	41 (36)	1.23 (0.73 to 2.08)	0.44	1.36 (0.76 to 2.44)	0.30
	Female	94 (69)	43 (31)	Reference		Reference	
Parent age	18-35 years	53 (62)	33 (38)	**2.49* (1.18 to 5.26)**	0.02	**2.90* (1.30 to 6.44)**	0.01
	36-45 years	62 (62)	38 (38)	**2.45* (1.18 to 5.09)**	0.02	**2.43* (1.12 to 5.28)**	0.03
	46 years ≥	52 (80)	13 (20)	Reference		Reference	
Parent living region	North of England	46 (70)	20 (30)	0.80 (0.39 to 1.63)	0.54	1.04 (0.49 to 2.22)	0.92
	Midlands England	42 (65)	23 (35)	1.01 (0.50 to 2.03)	0.98	1.03 (0.49 to 2.19)	0.93
	Wales, Scotland, Northern Ireland	31 (76)	15 (33)	0.89 (0.41 to 1.95)	0.78	1.15 (0.50 to 2.63)	0.75
	South of England	48 (65)	26 (35)	Reference		Reference	
Parent household income	≤ £34,999	68 (67)	34 (33)	0.95 (0.55 to 1.63)	0.84	1.04 (0.55 to 1.95)	0.91
	£35,000 ≥	87 (65)	46 (35)	Reference		Reference	
Parent employment status §	Working	131 (66)	69 (35)	1.26 (0.65 to 2.47)	0.49	1.25 (0.56 to 2.77)	0.59
	Not working	36 (71)	15 (29)	Reference		Reference	
Parent education level	≤ A-level	88 (69)	40 (31)	0.82 (0.48 to 1.38)	0.45	0.92 (0.51 to 1.64)	0.77
	Degree ≥	79 (64)	44 (36)	Reference		Reference	
My child has missed too much school since September this year ‡	5-point Likert-scale (1=strongly agree, 5=strongly disagree)	N=166, M=3.11, SD=1.48	N= 83, M=3.17, SD=1.54	1.02 (0.86 to 1.22)	0.79	1.03 (0.85 to 1.26)	0.74
My child is behind at school ‡	5-point Likert-scale (1=strongly agree, 5=strongly disagree)	N=167, M=3.65, SD=1.35	N=84, M=3.21, SD=1.57	**0.81* (0.67 to 0.97)**	0.02	0.84 (0.69 to 1.02)	0.08
My child often says they have symptoms of illnesses when they do not ‡	5-point Likert-scale (1=strongly agree, 5=strongly disagree)	N= 167, M=3.89, SD=1.25	N=84, M=3.54, SD=1.49	**0.83* (0.68 to 0.99)**	0.05	0.84 (0.67 to 1.05)	0.12
Often no-one is available to look after my child if they cannot go to school ‡	5-point Likert-scale (1=strongly agree, 5=strongly disagree)	N=166, M=3.46, SD=1.44	N= 84, M=3.11, SD=1.55	0.85 (0.71 to 1.02)	0.07	0.88 (0.72 to 1.07)	0.20
My child does not want to take time off school ‡	5-point Likert-scale (1=strongly agree, 5=strongly disagree)	N= 162, M=2.43, SD=1.17	N=83, M=2.11, SD=1.14	**0.78* (0.61 to 0.99)**	0.04	0.82 (0.63 to 1.05)	0.12

For peer review only - http://bmjopen.bmj.com/site/about/guidelines.xhtml

My child makes their own decisions about when they go to school ‡	5-point Likert-scale (1=strongly agree, 5=strongly disagree)	N=166, M=4.25, SD=0.98	N=83, M=3.60, SD=1.50	**0.65** (0.52 to 0.81)**	<0.001	**0.65** (0.50 to 0.83)**	<0.001
My child should go to school if they have taken medication (e.g., Calpol, paracetamol) ‡	5-point Likert-scale (1=strongly agree, 5=strongly disagree)	N=164, M=3.04, SD=1.30	N=81, M=2.25, SD=1.20	**0.60** (0.48 to 0.76)**	<0.001	**0.57** (0.44 to 0.75)**	<0.001
If children have common illnesses (e.g., a cold), they should go to school ‡	5-point Likert-scale (1=strongly agree, 5=strongly disagree)	N=166, M=2.30, SD=1.09	N=83, M=2.13, SD=1.03	0.86 (0.66 to 1.11)	0.24	0.83 (0.63 to 1.09)	0.19
Children build up their immunity by mixing with children who have common illnesses (e.g., a cold) ‡	5-point Likert-scale (1=strongly agree, 5=strongly disagree)	N=164, M=1.93, SD=0.88	N=84, M=1.90, SD=1.03	0.97 (0.73 to 1.29)	0.86	0.92 (0.67 to 1.26)	0.61
Other children with common illnesses (e.g., a cold) go to school ‡	5-point Likert-scale (1=strongly agree, 5=strongly disagree)	N=162, M=1.88, SD=0.79	N=83, M=1.84, SD=0.99	0.96 (0.70 to 1.30)	0.77	0.91 (0.65 to 1.27)	0.58
If children have mild symptoms of an illness, they should go to school ‡	5-point Likert-scale (1=strongly agree, 5=strongly disagree)	N=162, M=1.88, SD=0.79	N=80, M=2.46, SD=1.00	**0.77* (0.59 to 0.99)**	0.04	**0.75* (0.56 to 0.99)**	0.05
Going to school is important for my child's mental health ‡	5-point Likert-scale (1=strongly agree, 5=strongly disagree)	N=164, M=1.45, SD=0.67	N=83, M=1.66, SD=0.93	**1.41* (1.01 to 1.97)**	0.04	1.43 (0.99 to 2.06)	0.06
When my child says they are too ill to attend school, I let them stay at home ‡	5-point Likert-scale (1=strongly agree, 5=strongly disagree)	N=161, M=2.58, SD=0.67	N=84, M=2.56, SD=1.15	0.98 (0.77 to 1.25)	0.87	0.96 (0.74 to 1.25)	0.78

*P≤ 0.05 and formatted bold
**P≤ 0.001 and formatted bold.
† Odds ratio adjusted by parent gender, age, living region, household income, employment status, and education level
‡ The exact wording used in the survey
§Working includes students and volunteers.
Abbreviations: N = number of observations; M = mean; SD = standard deviation

For peer review only - http://bmjopen.bmj.com/site/about/guidelines.xhtml

Table 2 Children who engaged in each activity that we reported about when they had stay-at-home symptoms (n = 251)

Exact statement wording, in the order that they were asked to participants.	Children engaged in activity ("non-adherent"), n (%)	Children did not engage activity ("adherent"), n (%)
Went to school	60 (24%)	191 (76%)
Went to a club or lesson outside of school	29 (12%)	222 (88%)
Visited someone from another household	27 (11%)	224 (89%)
Someone from another household visited the child	15 (6%)	236 (94%)
Someone from another household visited our household	24 (10%)	227 (90%)

*Column totals do not add to 100% as participants could tick multiple options.

DISCUSSION

We found that 33% of children were non-adherent to UK Government guidance, such as attending school or clubs or socialising with others when experiencing symptoms where it is advised to stay at home. These findings align with previous reports suggesting that this behaviour, known as presenteeism, is common [12-14]. We also found that non-adherence was more likely for children with younger parents and for specific parental attitudes about symptoms of illness and their children's behaviour. This is concerning because children who attend school and socialise whilst unwell risk spreading their illness to others, increasing the likelihood that other children will need to take time off school and miss out on extra-curricular and social activities, and putting vulnerable people that they come into contact with at risk.

We found that children were significantly more likely to be non-adherent when their parents believed that children should go to school when their symptoms were mild. This finding mirrors Woodland, et al. [12] and Webster, et al. [15] who found people were more likely to attend school or work with an infectious illness when they perceived their symptoms to be mild. We also found that children were more likely to be non-adherent if a parent felt that attendance at school was appropriate if analgesic medication had been taken. These findings suggest that a key consideration is whether the child will cope with school, rather than whether they might spread illness to others. These findings align with previous research that suggested families were less likely to self-isolate with symptoms that may indicate COVID-19 when parents, a) perceived the symptoms to be mild, b) were unsure of the cause of symptoms and c) perceived a reduced severity of symptoms after taking analgesic medication [24]. A greater focus on the importance of decreasing social mixing when symptomatic may help reduce the incidence and spread of infectious disease outbreaks in schools and to others in the community.

For peer review only - http://bmjopen.bmj.com/site/about/guidelines.xhtml

Children were significantly more likely to be non-adherent where their parent reported that the child was responsible for making their own decisions about when they go to school. This mirrors the suggestion that children are more important in decision-making than previously recognised [12]. Educating children, particularly teenagers who may have a greater say over their attendance, about the need to remain at home when ill may be an important part of any future strategy to reduce presenteeism. But this finding may need to be taken with caution; the data were collected in the autumn term when schools re-opened following national school closures due to the pandemic. Reports show that children were keen to get back to school at this time, which may have impacted this result [25]. Still, educating parents on these issues is also important. We found that parents who were aged 45 years or younger were significantly more likely to have a child that was non-adherent. We can presume that these parents have younger children, and therefore we expect parents to have more control over their child's activities compared to older children. While younger parents may rely on more support from outside the household with childcare, in contrast to previous findings [15]. We did not find a significant association between children who were non-adherent and whether parents had someone available to look after their child if they could not go to school [12,15].

We were surprised that we did not find a significant association between children who were non-adherent and parents who felt their child had missed too much school and were behind in school. This was also replicated in our sensitivity analyses, that focused on children who had attended school and excluded all other types of non-adherent. Webster, et al. [15] found that people who had missed too much work and were concerned about their workload were more likely to attend work with an infectious illness. Woodland, et al. [12] also found that high motivations about school and high school absence were found to increase the risk of school-based presenteeism. We cannot be sure why our results contrast with previous research, but this could be due to the differences between parents not attending work (e.g., financial implications) and children not attending school (e.g., educational impacts) and pandemic-related reasons (e.g., increased access to online schooling and activities).

We found no significant associations between gender of the parental participant and reports of a child's adherence.[12] Unfortunately, as children's demographic data were not collected, we were unable to explore associations with this variable in the study or other child demographic characteristics that may have an impact on our findings. For example, it has been suggested that children who are in transition years are at increased risk of school-based presenteeism [12]. Children need to be included in future studies about school-based presenteeism as it is common for only the parents to be the participants [12].

There were several limitations to this study. On average 69% of children report at least one episode of school-based presenteeism [12], which is higher than our finding of 33%. However, this average was taken from three studies, two of which measured presenteeism over 12 months whereas ours was assessed for less than 4 months. In addition, we asked parents to report about their child's most severe episode of recent illness and we only reported non-adherence for one child from

For peer review only - http://bmjopen.bmj.com/site/about/guidelines.xhtml

each household for the four months that we measured. As such, we may have underestimated the prevalence of non-adherence: children who engaged in multiple bouts of non-adherence and households in which multiple children engaged in non-adherence will not have been identified. We also had a relatively small sample size and therefore we may not have detected small effects [26]. Our findings not only report on presenteeism behaviour but also interacting with others while symptomatic, therefore caution must be taken when comparing our findings to studies solely reporting on school-based presenteeism.

In addition, the data were drawn from a non-probability sample. Whether the sample was behaviourally and psychologically representative of the wider population of parents in the UK is unknown [27], although we have no reason to suspect that the associations within the data cannot be generalised [28]. Our participants were mainly of white ethnicity (91%, n=224/246, n=5 were missing data) and we were unable to include ethnicity in our analysis due to small case numbers. Although this is fairly representative of the UK population, we were unable to compare results across different ethnic groups. We did not include child demographics in our analysis due to survey space limitations thus we were unable to identify how children's characteristics may impact non-adherence. Our data collection occurred during a pandemic, with a substantial focus on the importance of remaining home when potentially infectious and after an extended period of home-schooling. How rates and predictors of presenteeism will change as we emerge from the pandemic is unknown.

In conclusion, one-third of children with symptoms that indicated an infectious illness engaged in activities outside the home, including going to school or socialising with others. This behaviour goes against UK Government advice that is in place to prevent the spread of disease. We found that younger parents, children who made their own decisions about school attendance, children who had taken analgesic medication and having mild symptoms were risk factors for non-adherence. We suggest that parents and teenagers may benefit from guidance to help them make informed decisions about school attendance whilst unwell to reduce school presenteeism. Given, our study had a small sample size and was conducted during a pandemic, it is recommended that further research is needed to validate our findings in non-pandemic times and to identify possible interventions that may be generalisable to general circulating and seasonal infections.

Data availability statement

The data set and survey questions related to this study can be accessed via the King's College London research data repository, KORDS (DOI 10.18742/19519444). The larger five-wave dataset will be archived with the UK Data Service after the end of the project, reference ES/V015494/1. This dataset can be matched with the full dataset on the ID variable.

Author contributions

All authors contributed to the conceptualisation of the study and approved the final draft. LW designed the survey, analysed the data and drafted the manuscript. LES,

For peer review only - http://bmjopen.bmj.com/site/about/guidelines.xhtml

RA and GJR designed the survey and edited the manuscript. RKW edited the manuscript. RKW, RA and GJR supervised LW.

Funding statement

This study was funded by the Economic and Social Research Council [grant number ES/P000703/1] and by the National Institute for Health and Care Research Health Protection Research Unit (NIHR HPRU) in Emergency Preparedness and Response [grant number NIHR200890], a partnership between the UK Health Security Agency, King's College London and the University of East Anglia. The views expressed are those of the author(s) and not necessarily those of the NIHR, UKHSA or the Department of Health and Social Care. For the purpose of open access, the author has applied a Creative Commons Attribution (CC BY) licence to any Author Accepted Manuscript version arising.

The funders had no role in study design, data collection, data analysis, data interpretation, or writing of the manuscript. The corresponding author had full access to all the data and had final responsibility for the decision to submit for publication.

Conflict of interest disclosure

GJR, RA and LS participate in the UK's Scientific Advisory Group for Emergencies, or its subgroups. These groups did not fund the study or authors. RA is an employee of the UK Health Security Agency. LW and RKW have no competing interests to declare.

REFERENCES

1. National Health Service. Is my child too ill for school? 2021 [updated 1 April. Available from: https://www.nhs.uk/live-well/healthy-body/is-my-child-too-ill-for-school/ accessed 3 February 2022.
2. HM Government. Health protection in schools and other childcare facilities 2021 [updated 3 March 2021. Available from: https://www.gov.uk/government/publications/health-protection-in-schools-and-other-childcare-facilities accessed 9 February 2022.
3. HM Government. Stay at home: guidance for households with possible or confirmed coronavirus (COVID-19) infection 2022 [updated 1 February. Available from: https://www.gov.uk/government/publications/covid-19-stay-at-home-guidance/stay-at-home-guidance-for-households-with-possible-coronavirus-covid-19-infection accessed 3 February 2022.
4. HM Government. Prime Minister's statement on coronavirus (COVID-19): 23 March 2020: HM Government,; 2020 [Available from: https://www.gov.uk/government/speeches/pm-address-to-the-nation-on-coronavirus-23-march-2020 accessed 11 June 2020.
5. HM Government. Norovirus outbreaks increasing in England, 2022.
6. Simon AK, Hollander GA, McMichael A. Evolution of the immune system in humans from infancy to old age. *Proceedings of the Royal Society B: Biological Sciences* 2015;282(1821):20143085.
7. Woodland L, Hodson A, Webster RK, et al. A Qualitative Study Evaluating the Factors Affecting Families' Adherence to the First COVID-19 Lockdown in England Using the COM-B Model and TDF. *International Journal of*

For peer review only - http://bmjopen.bmj.com/site/about/guidelines.xhtml

Environmental Research and Public Health 2022;19(12):7305. doi: 10.3390/ijerph19127305
8. Cleary V, Slaughter R, Heathcock R. An Infection Control Programme in Primary Schools and the Wider Public Health Impact. *Journal of Infection Prevention* 2003;4(5):11-17. doi: 10.1177/175717740300400503
9. Malosh RE, Martin ET, Ortiz JR, et al. The risk of lower respiratory tract infection following influenza virus infection: A systematic and narrative review. *Vaccine* 2018;36(1):141-47.
10. Thomson RM, Henderson HJ, Smith-Palmer A. An outbreak of Salmonella Saintpaul in a Scottish childcare facility: the influence of parental under-reporting. *BMC Infectious Diseases* 2019;19(1):847. doi: https://dx.doi.org/10.1186/s12879-019-4516-z
11. Joseph C, Noah N, White J, et al. A review of outbreaks of infectious disease in schools in England and Wales 1979–88. *Epidemiology and Infection* 1990;105(2):419-34. doi: 10.1017/s0950268800048007
12. Woodland L, Brooks SK, Webster RK, et al. Risk factors for school-based presenteeism in children: a systematic review. *BMC psychology* 2023;11(1):169.
13. Johansen V. Sick and still at school: an empirical study of sickness presence among students in Norwegian secondary school. *BMJ Open* 2015;5(9):e008290. doi: https://dx.doi.org/10.1136/bmjopen-2015-008290
14. BUPA. Bupa report reveals that half of all UK parents send children to school when ill 2010 [updated 26 October. Available from: https://pressreleases.responsesource.com/news/60199/bupa-report-reveals-that-half-of-all-uk-parents-send/ accessed 11 June 2020.
15. Webster RK, Liu R, Karimullina K, et al. A systematic review of infectious illness Presenteeism: prevalence, reasons and risk factors. *BMC Public Health* 2019;19(1):799. doi: 10.1186/s12889-019-7138-x
16. Ipsos MORI. About us n.d [Available from: https://www.ipsos.com/en-uk/about-us accessed 17 February 2022.
17. Ipsos MORI. Ipsos Answers Esomar's Questions to help online research buyers 2023 [updated 30 March. Available from: https://www.ipsos.com/en/ipsos-answers-esomars-questions-help-online-research-buyers accessed 17 July 2023.
18. PAMCo. PAMCo 4 2019 2019 [Available from: https://pamco.co.uk/2019/12/09/pamco-4-2019-data/ accessed 22 July 2023.
19. Office for National Statistics. Families and the labour market, UK: 2019 2019 [updated 24 October. Available from: https://www.ons.gov.uk/employmentandlabourmarket/peopleinwork/employmentandemployeetypes/articles/familiesandthelabourmarketengland/2019 accessed 10 March 2022.
20. Office for National Statistics. The effects of taxes and benefits on household income, disposable income estimate: financial year ending 2021 2023 [updated 25 January. Available from: https://www.ons.gov.uk/peoplepopulationandcommunity/personalandhouseholdfinances/incomeandwealth/datasets/householddisposableincomeandinequality accessed 22 July 2023.
21. von Elm E, Altman DG, Egger M, et al. The Strengthening the Reporting of Observational Studies in Epidemiology (STROBE) Statement: Guidelines for

For peer review only - http://bmjopen.bmj.com/site/about/guidelines.xhtml

Reporting Observational Studies. *Annals of Internal Medicine* 2007;147(8):573-77. doi: 10.7326/0003-4819-147-8-200710160-00010
22. IBM Corp. IBM SPSS Statistics for Windows, 28.0 Armonk, NY 2021 [
23. Office for National Statistics. Families and households 2023 [updated 18 May. Available from: https://www.ons.gov.uk/peoplepopulationandcommunity/birthsdeathsandmarriages/families/datasets/familiesandhouseholdsfamiliesandhouseholds accessed 17 July 2023.
24. Woodland L, Mowbray F, Smith LE, et al. What influences whether parents recognise COVID-19 symptoms, request a test and self-isolate: A qualitative study. *PLOS ONE* 2022;17(2):e0263537. doi: 10.1371/journal.pone.0263537
25. Children's Commissioner. Some sort of normal 2020 [updated 2 November. Available from: https://www.childrenscommissioner.gov.uk/education/ accessed 11 March 2022.
26. Button KS, Ioannidis JPA, Mokrysz C, et al. Power failure: why small sample size undermines the reliability of neuroscience. *Nature Reviews Neuroscience* 2013;14(5):365-76. doi: 10.1038/nrn3475
27. Epstein WM. Response bias in opinion polls and American social welfare. *The Social Science Journal* 2006;43(1):99-110. doi: 10.1016/j.soscij.2005.12.010
28. Kohler U. Possible Uses of Nonprobability Sampling for the Social Sciences. *Survey Methods: Insights from the Field* 2019 [updated 2 April. Available from: https://surveyinsights.org/?p=10981 accessed 29 July 2020.

For peer review only

For peer review only - http://bmjopen.bmj.com/site/about/guidelines.xhtml

Text S1: Survey questions

Standard demographic variables:

1. What is your date of birth?

1. YEAR
2. 1910 1910
3. ...
4. 2015 2015
5. MONTH
6. January
7. February
8. March
9. April
10. May
11. June
12. July
13. August
14. September
15. October
16. November
17. December

2. Which of the following describes how you think of yourself?

1. Male
2. Female
3. In another way
4. Prefer not to answer

3. Where do you live? Please note: This question may be considered personal. We would like to remind you that your participation is strictly voluntary and that your responses are used for research purposes only. The answers that you provide will be presented in aggregate form and none of them will be linked back to you in any way. All data will be collected and processed in adherence to the Market Research Society's Code of Conduct and the General Data Protection Regulation (GDPR).

1. Postcode
2. Postal Town
3. Prefer Not to Answer

4. Where do you live?

1. North East
2. North West
3. Yorkshire and The Humber
4. West Midlands
5. East Midlands
6. East of England

For peer review only

7. South West
8. South East
9. Greater London
10. Wales
11. Scotland
12. Northern Ireland

5. How many people are living or staying at your current address? (Include yourself and any other adults or children who are currently living or staying at this address for at least two months)

1. 1
2. 2
3. 3
4. 4
5. 5
6. 6
7. 7
8. 8
9. 9
10. 10
11. 11
12. 12+

6. How many children under the age of 18 are living in your household? Please reference only the children for which you are the parent or legal guardian. (If there are no children under 18 in your household, please type 0)

7. Please provide us with the following information about the children under the age of 18 in your household. Please reference only the children for which you are the parent or legal guardian.

1. Relationship
2. Parent (biological or adopted)
3. Legal guardian
4. Other (e.g. step-child)
5. Gender
6. Boy
7. Girl
8. Year of birth
9. Month of birth

8. What is your highest level of education attained?

1. Primary school
2. Secondary school (age under 15 years old)
3. GNVQ / GSVQ / GCSE/ SCE standard.
4. NVQ1, NVQ2
5. NVQ3/ SCE Higher Grade/ Advanced GNVQ/ GCE A/AS or similar.
6. NVQ4 / HNC / HND / Bachelor's degree or similar.
7. NVQ5 or post-graduate diploma.

For peer review only

For peer review only - http://bmjopen.bmj.com/site/about/guidelines.xhtml

Covid-19 Trust & Perceptions

November-December 2021

9. The next question may be considered personal, but it is not mandatory to answer. If you do, we assure you that your responses will be kept strictly confidential and used for research purposes only.   What is the COMBINED TOTAL ANNUAL INCOME (pre-tax) earned by all members of your household?   Please include all your income sources : salaries, scholarships, pension and Social Security benefits, dividends from shares, income from rental properties, child support and alimony etc.

1. Under £5,000
2. £5,000 - £9,999
3. £10,000 - £14,999
4. £15,000 - £19,999
5. £20,000 - £24,999
6. £25,000 - £34,999
7. £35,000 - £44,999
8. £45,000 - £54,999
9. £55,000 - £99,999
10. £100,000 or more
11. Prefer not to answer

10. What is your current employment status?

1. Employed full-time
2. Employed part-time
3. Self employed
4. Unemployed but looking for a job
5. Unemployed and not looking for a job/Long-term sick or disabled
6. Full-time parent, homemaker
7. Retired
8. Student/Pupil

11. In which of the below categories does your occupation fall? If retired or unemployed, please indicate the category closest to your previous occupation.

Responses copied from https://www.ilo.org/public/english/bureau/stat/isco/isco88/

12. What is the occupation of the person with the highest income? If retired or unemployed, please indicate the category closest to his/her previous occupation.

Responses copied from https://www.ilo.org/public/english/bureau/stat/isco/isco88/

In this survey, some questions will be about health and perceptions about, coronavirus, politics, religious beliefs, etc. A "Prefer not to answer" option will be available for you to select, if the case. Ipsos is running the survey and collecting such data in order to understand attitudes towards public health measures in the context of the coronavirus pandemic, and trust in their efficacy.

Ipsos has been commissioned by the University of Bristol and King's College London to carry out this research. Information collected through the survey will be made available to the team of researchers at the University of Bristol and King's College London in an anonymised format, unless you provide your permission to pass personal data to them. At

For peer review only - http://bmjopen.bmj.com/site/about/guidelines.xhtml

no point will any information that would allow you to be identified be made available to the public.

Participation is completely voluntary and you may withdraw your consent at any time. Your survey answers will be combined with the answers from all other participants and used for social research purposes only, and your personal data will be held for no longer than 12 months within Ipsos and no longer than April 2023 from researchers at the University of Bristol and King's College London.

Do you accept the collection of personal information and of health and perceptions about coronavirus related data?

1. *Yes, I accept*
2. *No, I do not accept*

13. Which of these applies to your home?

1. Being bought on a mortgage
2. Owned outright by household
3. Rented from a local authority
4. Rented from a housing association/trust
5. Rented from a private landlord
6. Other
7. Refused/don't know

The next question may be considered personal, but it is not mandatory to answer. If you do, we assure you that your responses will be kept strictly confidential and used for research purposes only.

14. What is your ethnic group?

Please select only one

White

1. English / Welsh / Scottish / Northern Irish / British
2. Irish
3. Gypsy or Irish Traveller
4. Any other White background

Mixed / multiple ethnic groups

5. White and Black Caribbean
6. White and Black African
7. White and Asian
8. Any other Mixed / multiple ethnic background

Asian / Asian British

9. Indian
10. Pakistani

For peer review only

11. Bangladeshi
12. Chinese
13. Any other Asian background

Black / African / Caribbean / Black British

14. African
15. Caribbean
16. Any other Black / African / Caribbean background

Other ethnic group

17. Arab
18. Any other ethnic group, please write in
19. Prefer not to answer

For peer review only

Survey questions

For the next set of questions, we would like you to think about your [number of children] youngest children who usually attend school.

15. Since *about* [Scotland only] the start of the school year (September 2021), please tick, for each child, any new symptoms that they developed for any reason (e.g. a cold, allergic reaction, coronavirus, etc.), even if some of the symptoms were mild. If they have developed new symptoms more than once during that period (for example if they have had a cold in one week, and an allergic reaction a couple of weeks later), please just tell us about the most severe set of symptoms that they had (for example, tell us about the cold, or the allergic reaction, whichever seemed worse). Please tick any that apply.

1. New, continuous cough
2. High temperature / fever
3. Diarrhoea
4. Nausea / feeling sick
5. Vomiting / being sick
6. Earache
7. A rash
8. Loss of sense of smell (fully or partial)
9. Loss of taste
10. Sore throat
11. Headache
12. Muscle ache
13. Breathlessness, tight chest, or wheezing
14. Feeling tired or exhausted
15. I don’t have any (more) children who usually attend school
16. None of these
17. Don’t know
18. Prefer not to say

For peer review only - http://bmjopen.bmj.com/site/about/guidelines.xhtml

1. Child 1 (youngest)
2. Child 2
3. Child 3
4. Child (oldest)

16. Please think about your child who most recently developed symptoms. When they had symptoms, did they do any of the following?
Please tick any that apply. Do not count things that they did online.

1. Went to school
2. Went to a club or lesson outside of school
3. Visited someone from another household
4. Someone from another household visited the child
5. Someone from another household visited our household
6. Took a lateral flow test (LFT) to see whether they had coronavirus (this is a rapid test that gives results in 30 minutes)
7. Took a polymerase chain reaction (PCR) test to see whether they had coronavirus (this is a test that is sent off to a laboratory)
8. Took both a lateral flow test (LFT, a rapid test that gives results in 30 minutes) and a polymerase chain reaction (PCR) test (a test that you send off to the laboratory) to see whether they had coronavirus
9. Took a test to see whether they had coronavirus, but I am not sure what type of test it was
10. None of these
11. Prefer not to say

17. Please still think about your child who most recently developed symptoms. You said your child had taken at least one rapid lateral flow test (LFT). Were any of the results positive?

1. Yes, this child had one or more rapid lateral flow tests (LFT) and had a positive result
2. None of the rapid lateral flow tests (LFT) for this child had a positive result
3. Don't know
4. Prefer not to say

18. Please still think about your child who most recently developed symptoms. You said your child had taken a polymerase chain reaction (PCR) test to see whether they had coronavirus. What was the result?

1. My child tested positive
2. My child tested negative
3. The result was inconclusive
4. I have not received the results
5. Don't know
6. Prefer not to say

For peer review only - http://bmjopen.bmj.com/site/about/guidelines.xhtml

19. Thinking about the same child who most recently developed symptoms, to what extent, if at all, do you agree or disagree with the following statements about this child, as a result of having these symptoms?

1. My child has missed too much school since September this year
2. My child is behind at school
3. My child often says they have symptoms of illnesses when they do not
4. Often no-one is available to look after my child if they cannot go to school
5. My child does not want to take time off school
6. My child makes their own decisions about when they go to school
7. My child should go to school if they have taken medication (e.g., Calpol, paracetamol)

    1. Strongly agree
    2. Tend to agree
    3. Neither agree nor disagree
    4. Tend to disagree
    5. Strongly disagree
    6. Don't know
    7. Prefer not to say

20. Now thinking in general, to what extent, if at all, do you agree or disagree with the following statements?

1. If children have common illnesses (e.g., a cold), they should go to school
2. Children build up their immunity by mixing with children who have common illnesses (e.g., a cold)
3. Other children with common illnesses (e.g. a cold) go to school
4. If children have mild symptoms of an illness, they should go to school
5. Going to school is important for my child's mental health
6. When my child says they are too ill to attend school, I let them stay at home

    1. Strongly agree
    2. Tend to agree
    3. Neither agree nor disagree
    4. Tend to disagree
    5. Strongly disagree
    6. Don't know
    7. Prefer not to say

For peer review only - http://bmjopen.bmj.com/site/about/guidelines.xhtml

Supplement 3: Checklist for Reporting Of Survey Studies (CROSS)

Section/topic	Item	Item description	Reported on page #
**Title and abstract**			
Title and abstract	1a	State the word "survey" along with a commonly used term in title or abstract to introduce the study's design. #2	
	1b	Provide an informative summary in the abstract, covering background, objectives, methods, findings/results, interpretation/discussion, and conclusions. #2	
**Introduction**			
Background	2	Provide a background about the rationale of study, what has been previously done, and why this survey is needed. #4	
Purpose/aim	3	Identify specific purposes, aims, goals, or objectives of the study. #4	
**Methods**			
Study design	4	Specify the study design in the methods section with a commonly used term (e.g., cross-sectional or longitudinal). #5	
Data collection methods	5a	Describe the questionnaire (e.g., number of sections, number of questions, number and names of instruments used).	
	5b	Describe all questionnaire instruments that were used in the survey to measure #5 particular concepts. Report target population, reported validity and reliability information, scoring/classification procedure, and reference links (if any). #5	
	5c	Provide information on pretesting of the questionnaire, if performed (in the article or in an online supplement). Report the method of pretesting, number of times questionnaire was pre-tested, number and demographics of participants used for pretesting, and the level of similarity of demographics between pre-testing participants and sample population. #5	
	5d	Questionnaire if possible, should be fully provided (in the article, or as appendices or as an online supplement). #this has been provided (Text S1 Survey)	
Sample characteristics	6a	Describe the study population (i.e., background, locations, eligibility criteria for participant inclusion in survey, exclusion criteria). #5	
	6b	Describe the sampling techniques used (e.g., single stage or multistage sampling, simple random sampling, stratified sampling, cluster sampling, convenience sampling). Specify the locations of sample participants whenever clustered sampling was applied. #5	
	6c	Provide information on sample size, along with details of sample size calculation.	
	6d	Describe how representative the sample is of the study population (or target population if possible), particularly for population-based surveys. #5	

For peer review only

Survey administration	7a	Provide information on modes of questionnaire administration, including the type and number of contacts, the location where the survey was conducted (e.g., outpatient room or by use of online tools, such as SurveyMonkey). #5
	7b	Provide information of survey's time frame, such as periods of recruitment, exposure, and follow-up days. #5
	7c	Provide information on the entry process: >For non-web-based surveys, provide approaches to minimize human error in data entry. >For web-based surveys, provide approaches to prevent "multiple participation" of participants. #5
Study preparation	8	Describe any preparation process before conducting the survey (e.g., interviewers' training process, advertising the survey). #5
Ethical considerations	9a	Provide information on ethical approval for the survey if obtained, including informed consent, institutional review board [IRB] approval, Helsinki declaration, and good clinical practice [GCP] declaration (as appropriate). #7
	9b	Provide information about survey anonymity and confidentiality and describe what mechanisms were used to protect unauthorized access. #7
Statistical analysis	10a	Describe statistical methods and analytical approach. Report the statistical software that was used for data analysis. #7
	10b	Report any modification of variables used in the analysis, along with reference (if available). #7
	10c	Report details about how missing data was handled. Include rate of missing items, missing data mechanism (i.e., missing completely at random [MCAR], missing at random [MAR] or missing not at random [MNAR]) and methods used to deal with missing data (e.g., multiple imputation). #7
	10d	State how non-response error was addressed. #5
	10e	For longitudinal surveys, state how loss to follow-up was addressed.
	10f	Indicate whether any methods such as weighting of items or propensity scores have been used to adjust for non-representativeness of the sample. #5
	10g	Describe any sensitivity analysis conducted. #7
**Results**		
Respondent characteristics	11a	Report numbers of individuals at each stage of the study. Consider using a flow diagram, if possible. #8
	11b	Provide reasons for non-participation at each stage, if possible. #8
	11c	Report response rate, present the definition of response rate or the formula used to calculate response rate. #NA
	11d	Provide information to define how unique visitors are determined. Report number of unique visitors along with relevant proportions (e.g., view proportion, participation proportion, completion proportion). #NA
Descriptive results	12	Provide characteristics of study participants, as well as information on potential confounders and assessed outcomes. #8

For peer review only

Main findings	13a	Give unadjusted estimates and, if applicable, confounder-adjusted estimates along with 95% confidence intervals and p-values. #10
	13b	For multivariable analysis, provide information on the model building process, model fit statistics, and model assumptions (as appropriate). #8
	13c	Provide details about any sensitivity analysis performed. If there are considerable amount of missing data, report sensitivity analyses comparing the results of complete cases with that of the imputed dataset (if possible). #NA
**Discussion**		
Limitations	14	Discuss the limitations of the study, considering sources of potential biases and imprecisions, such as non-representativeness of sample, study design, important uncontrolled confounders. #12
Interpretations	15	Give a cautious overall interpretation of results, based on potential biases and imprecisions and suggest areas for future research. #13
Generalizability	16	Discuss the external validity of the results. #13
**Other sections**		
Role of funding source	17	State whether any funding organization has had any roles in the survey's design, implementation, and analysis. #14
Conflict of interest	18	Declare any potential conflict of interest. #15
Acknowledgements	19	Provide names of organizations/persons that are acknowledged along with their contribution to the research. #14

For peer review only

Reporting checklist for cross sectional study.

Based on the STROBE cross sectional guidelines.

Instructions to authors

Complete this checklist by entering the page numbers from your manuscript where readers will find each of the items listed below.

Your article may not currently address all the items on the checklist. Please modify your text to include the missing information. If you are certain that an item does not apply, please write "n/a" and provide a short explanation.

Upload your completed checklist as an extra file when you submit to a journal.

In your methods section, say that you used the STROBE cross sectionalreporting guidelines, and cite them as:

von Elm E, Altman DG, Egger M, Pocock SJ, Gotzsche PC, Vandenbroucke JP. The Strengthening the Reporting of Observational Studies in Epidemiology (STROBE) Statement: guidelines for reporting observational studies.

		Reporting Item	Page Number
**Title and abstract**			
Title	#1a	Indicate the study's design with a commonly used term in the title or the abstract	1

For peer review only

Abstract	#1b	Provide in the abstract an informative and balanced summary of what was done and what was found	2
**Introduction**			
Background / rationale	#2	Explain the scientific background and rationale for the investigation being reported	4
Objectives	#3	State specific objectives, including any prespecified hypotheses	4
**Methods**			
Study design	#4	Present key elements of study design early in the paper	5
Setting	#5	Describe the setting, locations, and relevant dates, including periods of recruitment, exposure, follow-up, and data collection	5
Eligibility criteria	#6a	Give the eligibility criteria, and the sources and methods of selection of participants.	5
	#7	Clearly define all outcomes, exposures, predictors, potential confounders, and effect modifiers. Give diagnostic criteria, if applicable	5
Data sources / measurement	#8	For each variable of interest give sources of data and details of methods of assessment (measurement). Describe comparability of assessment methods if there is more than one group. Give information separately for for exposed and unexposed groups if applicable.	5,6

For peer review only

Bias	#9	Describe any efforts to address potential sources of bias	5
Study size	#10	Explain how the study size was arrived at	5
Quantitative variables	#11	Explain how quantitative variables were handled in the analyses. If applicable, describe which groupings were chosen, and why	6
Statistical methods	#12a	Describe all statistical methods, including those used to control for confounding	7
Statistical methods	#12b	Describe any methods used to examine subgroups and interactions	6
Statistical methods	#12c	Explain how missing data were addressed	7
Statistical methods	#12d	If applicable, describe analytical methods taking account of sampling strategy	n/a
Statistical methods	#12e	Describe any sensitivity analyses	7
**Results**			
Participants	#13a	Report numbers of individuals at each stage of study—eg numbers potentially eligible, examined for eligibility, confirmed eligible, included in the study, completing follow-up, and analysed. Give information separately for for exposed and unexposed groups if applicable.	8
Participants	#13b	Give reasons for non-participation at each stage	8

For peer review only

Participants	#13c	Consider use of a flow diagram	n/a
Descriptive data	#14a	Give characteristics of study participants (eg demographic, clinical, social) and information on exposures and potential confounders. Give information separately for exposed and unexposed groups if applicable.	8
Descriptive data	#14b	Indicate number of participants with missing data for each variable of interest	10,11
Outcome data	#15	Report numbers of outcome events or summary measures. Give information separately for exposed and unexposed groups if applicable.	10,11
Main results	#16a	Give unadjusted estimates and, if applicable, confounder-adjusted estimates and their precision (eg, 95% confidence interval). Make clear which confounders were adjusted for and why they were included	10,11
Main results	#16b	Report category boundaries when continuous variables were categorized	10,11
Main results	#16c	If relevant, consider translating estimates of relative risk into absolute risk for a meaningful time period	n/a
Other analyses	#17	Report other analyses done—e.g., analyses of subgroups and interactions, and sensitivity analyses	9
**Discussion**			
Key results	#18	Summarise key results with reference to study objectives	13

For peer review only

Limitations	#19	Discuss limitations of the study, taking into account sources of potential bias or imprecision. Discuss both direction and magnitude of any potential bias.	14,15
Interpretation	#20	Give a cautious overall interpretation considering objectives, limitations, multiplicity of analyses, results from similar studies, and other relevant evidence.	14,15
Generalisability	#21	Discuss the generalisability (external validity) of the study results	14,15
**Other Information**			
Funding	#22	Give the source of funding and the role of the funders for the present study and, if applicable, for the original study on which the present article is based	15

The STROBE checklist is distributed under the terms of the Creative Commons Attribution License CC-BY. This checklist was completed on 03. January 2023 using https://www.goodreports.org/, a tool made by the EQUATOR Network in collaboration with Penelope.ai

For peer review only

Why do children attend school, engage in other activities, or socialise when they have symptoms of an infectious illness? A cross-sectional survey

Journal:	*BMJ Open*
Manuscript ID	bmjopen-2023-071599.R2
Article Type:	Original research
Date Submitted by the Author:	07-Sep-2023

Complete List of Authors:	Woodland, Lisa; King's College London, Psychological Medicine<br>Smith, Louise; King's College London, Psychological Medicine<br>Webster, Rebecca; The University of Sheffield, Psychology<br>Amlôt, Richard; UK Health Security Agency<br>Rubin, G James; King's College London, Psychological Medicine

**Primary Subject Heading**:	Health policy
Secondary Subject Heading:	Infectious diseases
Keywords:	INFECTIOUS DISEASES, COVID-19, Health policy < HEALTH SERVICES ADMINISTRATION & MANAGEMENT

SCHOLARONE™ Manuscripts

For peer review only - http://bmjopen.bmj.com/site/about/guidelines.xhtml

I, the Submitting Author has the right to grant and does grant on behalf of all authors of the Work (as defined in the below author licence), an exclusive licence and/or a non-exclusive licence for contributions from authors who are: i) UK Crown employees; ii) where BMJ has agreed a CC-BY licence shall apply, and/or iii) in accordance with the terms applicable for US Federal Government officers or employees acting as part of their official duties; on a worldwide, perpetual, irrevocable, royalty-free basis to BMJ Publishing Group Ltd ("BMJ") its licensees and where the relevant Journal is co-owned by BMJ to the co-owners of the Journal, to publish the Work in this journal and any other BMJ products and to exploit all rights, as set out in our licence.

The Submitting Author accepts and understands that any supply made under these terms is made by BMJ to the Submitting Author unless you are acting as an employee on behalf of your employer or a postgraduate student of an affiliated institution which is paying any applicable article publishing charge ("APC") for Open Access articles. Where the Submitting Author wishes to make the Work available on an Open Access basis (and intends to pay the relevant APC), the terms of reuse of such Open Access shall be governed by a Creative Commons licence – details of these licences and which Creative Commons licence will apply to this Work are set out in our licence referred to above.

Other than as permitted in any relevant BMJ Author's Self Archiving Policies, I confirm this Work has not been accepted for publication elsewhere, is not being considered for publication elsewhere and does not duplicate material already published. I confirm all authors consent to publication of this Work and authorise the granting of this licence.

For peer review only

Why do children attend school, engage in other activities, or socialise when they have symptoms of an infectious illness? A cross-sectional survey

Lisa Woodland MSc,[1,4*] Weston Education Centre, Cutcombe Road, London SE5 9RJ, UK (PhD Researcher in Psychological Medicine)

Louise E Smith PhD,[1,4] Weston Education Centre, Cutcombe Road, London SE5 9RJ, UK (Research Associate in Psychological Medicine)

Rebecca K Webster PhD,[2] Cathedral Court, 1 Vicar Lane, Sheffield, S1 2LT, UK (Lecturer, Department of Psychology)

Richard Amlôt PhD,[3,4] Behavioural Science and Insights Unit (BSIU), Porton Down, Salisbury, SP4 0JG, UK (Deputy Director, Behavioural Science and Insights Unit (BSIU))

G James Rubin PhD,[1,4] Weston Education Centre, Cutcombe Road, London SE5 9RJ, UK (Professor of Psychology & Emerging Health Risks)

[1] King's College London

[2] University of Sheffield

[3] UK Health Security Agency

[4] NIHR Health Protection Research Unit in Emergency Preparedness and Response

Correspondence to:

Lisa Woodland
King's College London, Weston Education Centre, Cutcombe Road, London SE5 9RJ, UK
lisa.woodland@kcl.ac.uk

Word count: 4,571

For peer review only - http://bmjopen.bmj.com/site/about/guidelines.xhtml

Keywords: Infectious diseases; non-adherence; children; presenteeism; COVID-19; diarrhoea; vomiting

For peer review only

For peer review only - http://bmjopen.bmj.com/site/about/guidelines.xhtml

ABSTRACT

Objectives

To prevent the spread of infectious disease, children are typically asked not to attend school, clubs or other activities, or socialise with others whilst they have specific symptoms. Despite this, many children continue to participate in these activities whilst symptomatic.

Design and setting

We commissioned a national cross-sectional survey with data collected between 19 November 19 and December 18, 2021.

Participants

Eligible parents (n=941) were between 18 and 75 years of age, lived in the UK and had at least one child aged between four and 17 years old. Parents were recruited from a pre-existing pool of potential respondents who had already expressed in interest in receiving market research surveys.

Outcome measures

Parents were asked whether their children had exhibited either recent vomiting, diarrhoea, high temperature/fever, a new continuous cough, a loss or change to their sense of taste or smell in the absence of a negative (PCR) COVID-19 test ("stay-at-home symptoms") since September 2021 and whether they attended school, engaged in other activities outside the home or socialised with members of another household whilst symptomatic ("non-adherent"). We also measured parent's demographics and attitudes about illness.

Results

One-third (33%, n=84/251, 95% CI 28% to 39%) of children were "non-adherent" in that they had attended activities outside the home or socialised when they had stay-at-home symptoms. Children were significantly more likely to be non-adherent when parents were aged 45 and younger; they allowed their children to make their own decisions about school attendance; they agreed that their child should go to school if they took over-the-counter medication; or they believed that children should go to school if they have mild symptoms of illness.

Conclusion

To reduce the risk of spreading disease, parents and teenagers need guidance to help them make informed decisions about engaging in activities and socialising with others whilst unwell.

For peer review only

STRENGTHS AND LIMITATIONS OF THIS STUDY

- A strength of the study is that we investigated reported behaviour, rather than intentions.
- The study used a non-probability sample, thus there is a risk of some sampling bias.
- Data about children's demographics were not collected, which limits our ability to interpret the data.

For peer review only

INTRODUCTION

In order to reduce the spread of infectious illness within schools, children who have specific symptoms, including fever, diarrhoea and vomiting, are commonly advised to remain at home [1,2]. While this has been the case for many years, this message was made more urgent by the COVID-19 pandemic. In the UK, throughout most of 2020 and 2021, anyone with a new continuous cough, a high temperature, or a loss or change to their sense of taste or smell were asked not to attend school or work nor to interact with people outside their household unless they had a negative COVID-19 polymerase chain reaction (PCR) test result [3,4]. This message is also important for other symptoms of infectious illnesses. For instance, in February 2022, cases of norovirus were 48% higher than expected in educational settings [5], resulting in a warning to the public about the health threat [5]. Children are particularly susceptible to infections because their immune systems are developing [6], they are often in close contact with other children, and although they can adhere to hygiene practices [7], they are at risk of infections [2,8,9]. Preventing ill children from mixing with others is essential in mitigating the spread of infection [10,11]. The UK Government has issued guidance on how long a child should not attend school for when they show symptoms of an infectious illness [2].

A systematic review indicates that a large proportion of symptomatic children may be attending school (known as “school-based presenteeism”) [12]. For example, one survey of 3,040 secondary school pupils in Norway reported that 58% had attended school in the past year despite feeling so ill that they should have stayed at home [13]. One in six British parents reported that they would send their child to school even if they were currently experiencing diarrhoea or vomiting [14]. The systematic review identified five themes that were shown to impact school-based presenteeism: children’s characteristics, children’s and parents’ motivations and attitudes towards school; organisational factors and school sickness policy [12]. Another systematic review found similar findings and suggested that people are more likely to attend school or work with symptoms of an infectious illness if they: are unsure of the sickness guidelines; are worried about disciplinary action; are unable to find alternative child care; are concerned about their workload; feel they have missed too much work; perceive a culture of presenteeism in the organisation; and perceive their illness to be mild or non-infectious [15]. The studies included in the systematic reviews focus only on work or school-based presenteeism and the findings in relation to children in the UK are limited. However, hypotheses can be drawn; and children who have positive motivations towards school, worry about school attendance and when the symptoms of illness are mild, will be more at risk of non-adherence.

In this study, we aimed to identify the proportion of children who were non-adherent (e.g., attended school, clubs, or other activities or who socialised with people outside their household) while they had symptoms that should require them to remain at home. We also aimed to identify the possible parent and child-level risk factors for engaging in these activities. The results of which can then be used to inform interventions that target these risk factors as a means to prevent the spread of infectious diseases.

For peer review only - http://bmjopen.bmj.com/site/about/guidelines.xhtml

METHODS

Design

We used data from a longitudinal study about perceptions of COVID-19, trust in public officials and other health attitudes and behaviours. We commissioned Ipsos MORI [16] to conduct this national cross-sectional online survey of 5,000 participants at each wave. This paper is based on data from wave 5, which collected data between November 19 and December 18, 2021.

Participants

Non-probability sampling was used for ease of participant recruitment and to limit study costs. Participants were recruited from a pre-existing pool of potential respondents who had already expressed an interest in receiving market research surveys. Participants were recruited by advertising via platforms such as social media, online gaming sites and news outlets. In Europe, 38% of the volume is recruited through social media, 26% through self-recruitment and referral and 36% through affiliate networks and media agencies. Further information about the company's recruitment process can be found in their guidance [17].

Participants aged between 18 and 75, and who lived in the UK were eligible for the survey. Quotas were set on the main sample: age interlocked by gender; government office region; working status; and social grade based on PAMCo 4 (October 2018 to September 2019) data[18]. One percent of participants were removed due to Ipsos MORI's quality control procedures [17]. The survey company perform automated quality checks, such as country geo-IP validation and removing duplicate email identifications. Further checks were made on the final dataset, and data that indicated inconsistencies, such as straight-lining, when the participant selects the same response throughout the survey, and incomplete surveys were removed.

Recruitment followed a complex pattern, in which responders from earlier waves were invited to participate and additional respondents from the market research panel were then invited to take part to take the place of non-responders. Response rates are not provided because they are not an accurate indicator of bias in quota samples—given that the underlying sample frame consists of people who have previously self-selected to receive invitations to take part in surveys, understanding whether the achieved sample (within each quota) is representative of the sample frame provides no information as to whether it is also representative of the wider general population.

Participants were paid between €1 and €1.50 for completing the survey.

The target population was parents of dependent children. As this was a sub-sample of a wider survey the participants and sample size were based on the needs for the main study, which set quotas to obtain a sample broadly representative of the UK adult population. In our parent sample: 80% were employed, in the UK 69% of parents are employed in lone parent households and both parents are employed in 74% of couple households [19]; 54% earned £35,000 and over and the average disposable income for UK households with children was £35,049 [20]; and 49% of

For peer review only - http://bmjopen.bmj.com/site/about/guidelines.xhtml

parents were highly educated, which is higher than the 34% of parents and non-parents in England and Wales [21].

Study materials

The wording of all survey items is available in the supplementary materials (Text S1).

Participant demographics

We asked parents to report their gender, age, postcode (to derive region), household income, employment status, ethnicity, and level of education. A non-response option was available for each of these demographic questions ("prefer not to say," "prefer not to answer," and for gender only, parents could also respond "in another way"). Due to space limitations in the survey, we did not collect child demographic data.

Identification of children with symptoms of illness

Parents were asked to consider their four (or fewer) youngest children aged between four and 17 years and to report for each child any symptoms they had experienced since the start of the school year (September 2021). We restricted the number of children parents could report on to four to allow most parents to report the symptoms for all of their children whilst limiting the length of the survey. In Scotland, the academic year started in August 2021, therefore, participants in Scotland were asked about their child's symptoms since "*about* the start of the school year (September 2021)". If a child had experienced multiple bouts of illness during that period, we asked parents to report the one set of symptoms they perceived as most severe. Parents were asked to report all symptoms that applied to their child from a list of 14 symptoms of infectious illnesses and ailments common in children, listed by the UK Government [2].

Children's activities whilst symptomatic

Parents who had at least one symptomatic child were asked to consider the child who most recently exhibited symptoms and to report whether, when they had symptoms, they engaged in any of the following activities (excluding online): going to school; going to a club or lesson outside of school; visiting someone from another household; having someone from another household visit them; having someone from another household visit the household in general. Parents were asked to "tick any [activity] that applied," or they could respond "none of these" or "prefer not to say."

We also asked parents whether their child had taken a COVID-19 test (lateral flow (LFT) test or a PCR test) while symptomatic, and if so, the result of the test.

Parent attitudes about their child and perceptions about illness

We asked parents 13 statements about their attitudes concerning their child (e.g., my child has missed too much school since September this year) and about common perceptions about illness (e.g., other children with common illnesses (e.g., a cold) go to school). We asked parents to respond to each statement on a five-point Likert scale from "strongly agree" to "strongly disagree"; they could also respond "don't know" or "prefer not to say."

For peer review only - http://bmjopen.bmj.com/site/about/guidelines.xhtml

Analysis

Analysis was conducted in SPSS [22]. We created binary and multinominal variables for parent demographics (presented in Table 1).

We recoded symptoms that necessitated the child to “stay-at-home” according to Government guidance as being: a new, continuous cough or a loss or change to their sense of taste or smell in the absence of a negative PCR result; a high temperature; vomiting; and diarrhoea. We restricted our analyses to parents of a child who had exhibited one or more of these stay-at-home symptoms.

We created a single binary variable indicating whether the child had attended at least one activity outside the home (except to get a PCR or LFT COVID-19 test), which included any interaction (inside or outside the home) with someone from another household (“non-adherence”). Children who had stayed at home (except to get a PCR or LFT COVID-19 test) and had not interacted with someone from another household were categorised as adherent.

For all variables, we coded the responses “in another way,” “prefer not to say/answer,” and “don’t know” as missing data. Participants had to complete each question before moving onto the next; as such there were no other missing data.

We ran separate binary logistic regressions to test univariable associations between children’s adherence and: parents’ demographic variables, attitudes about their child and perceptions about illness (as continuous variables). We ran a second set of binary logistic regressions adjusting for parent gender, age, region, income, employment status, and education level. The potential cofounders were entered into the regression model at the same time for every test.

We also re-ran sensitivity analyses investigating associations with school attendance only (excluding all other out-of-home activities and social interactions). These analyses were to identify differences in associations between parents of children who were non-adherent in any behaviour compared to children who were non-adherent due to only attending school.

Ethics

The research was approved by the Faculty of Social Sciences and Law, University of Bristol (approval code 116976 and 8273). Participants provided informed consent. Data were anonymised and only researchers related to this study had access.

Reporting

We used the STROBE cross sectional checklist when writing this manuscript [23].

Patient and public involvement

None.

RESULTS

For peer review only - http://bmjopen.bmj.com/site/about/guidelines.xhtml

4,962 participants completed the main survey, of whom 941 (19%) indicated that they had a child aged between four and 17 years. This proportion of respondents is as expected as 22% of households in the UK have a dependent child [24].

Of these 941 parents, 251 reported that their child had experienced symptom(s) that required them to stay at home.

Parents (n=941) reported on symptom(s) for 1,533 children aged four to 17 years. 48% of parents (n=454, 95% CI 45% to 51%) reported that at least one of their children had experienced at least one symptom and 27% (n=251, 95% CI 24% to 30%) reported that at least one child had experienced at least one stay-at-home symptom. Of the 251 children who had at least one stay-at-home symptom, 33% (n=84, 95% CI 28% to 39%) were non-adherent.

Most parents included in this study were female (55%, n = 137), aged between 36 and 45 years (40%, n = 100) and earned over £35,000 (57%, n = 133) (males: 45%, n = 114; aged 18 to 35 years: 34%, n = 86, aged over 46 years: 26%, n = 65; earned under £34,999: 43%, n = 102). Table 1 presents associations between predictor variables and whether children went to school, engaged in other activities, or socialised with non-household members (non-adherent). Non-adherence was moderately associated with younger parent age, with the odds of a child being non-adherent 2.9 times higher if the parent was aged 18-35, and 2.4 times higher if the parent was aged 36-45 compared to parents aged 46 or over.

Non-adherence was associated with parents' agreement with the following statements: their children made their own decisions about when to go to school; children should go to school if they take medication; and if children have a mild symptom of illness, they should go to school. Table 1 reports the exact statement wording, with lower values reflecting agreement with that statement.

Table 2 shows the frequencies of children's adherence and non-adherence for each of the behaviours that were then grouped into our outcome variable.

When re-running the analyses to look at associations with school attendance only (sensitivity analysis), 227 parents were included. Fewer participants were included in the sensitivity analysis because not all 251 children attended school while symptomatic with stay-at-home symptoms. We found school attendance was not associated with parent age, but it was associated with parents agreeing that their child did not want to take time off school. No other differences in associations were found between school attendance and non-adherence and our predictor variables (data not shown).

For peer review only - http://bmjopen.bmj.com/site/about/guidelines.xhtml

Table 1. Associations between parent demographics and attitudes, and whether their child (4 to 17 years, with at least one stay-at-home symptom) were non-adherent (n=251)

Parent demographics and attitudes about sending children to school whilst symptomatic	Level	Children were adherent (%)	Children were non-adherent (%)	Odds ratio (95% CI)	P-value	Adjusted odds ratio (95% CI) †	P-Value
Parent gender	Male	73 (64)	41 (36)	1.23 (0.73 to 2.08)	0.44	1.36 (0.76 to 2.44)	0.30
	Female	94 (69)	43 (31)	Reference		Reference	
Parent age	18-35 years	53 (62)	33 (38)	**2.49* (1.18 to 5.26)**	0.02	**2.90* (1.30 to 6.44)**	0.01
	36-45 years	62 (62)	38 (38)	**2.45* (1.18 to 5.09)**	0.02	**2.43* (1.12 to 5.28)**	0.03
	46 years ≥	52 (80)	13 (20)	Reference		Reference	
Parent living region	North of England	46 (70)	20 (30)	0.80 (0.39 to 1.63)	0.54	1.04 (0.49 to 2.22)	0.92
	Midlands England	42 (65)	23 (35)	1.01 (0.50 to 2.03)	0.98	1.03 (0.49 to 2.19)	0.93
	Wales, Scotland, Northern Ireland	31 (76)	15 (33)	0.89 (0.41 to 1.95)	0.78	1.15 (0.50 to 2.63)	0.75
	South of England	48 (65)	26 (35)	Reference		Reference	
Parent household income	≤ £34,999	68 (67)	34 (33)	0.95 (0.55 to 1.63)	0.84	1.04 (0.55 to 1.95)	0.91
	£35,000 ≥	87 (65)	46 (35)	Reference		Reference	
Parent employment status §	Working	131 (66)	69 (35)	1.26 (0.65 to 2.47)	0.49	1.25 (0.56 to 2.77)	0.59
	Not working	36 (71)	15 (29)	Reference		Reference	
Parent education level	≤ A-level	88 (69)	40 (31)	0.82 (0.48 to 1.38)	0.45	0.92 (0.51 to 1.64)	0.77
	Degree ≥	79 (64)	44 (36)	Reference		Reference	
My child has missed too much school since September this year ‡	5-point Likert-scale (1=strongly agree, 5=strongly disagree)	N=166, M=3.11, SD=1.48	N= 83, M=3.17, SD=1.54	1.02 (0.86 to 1.22)	0.79	1.03 (0.85 to 1.26)	0.74
My child is behind at school ‡	5-point Likert-scale (1=strongly agree, 5=strongly disagree)	N=167, M=3.65, SD=1.35	N=84, M=3.21, SD=1.57	**0.81* (0.67 to 0.97)**	0.02	0.84 (0.69 to 1.02)	0.08
My child often says they have symptoms of illnesses when they do not ‡	5-point Likert-scale (1=strongly agree, 5=strongly disagree)	N= 167, M=3.89, SD=1.25	N=84, M=3.54, SD=1.49	**0.83* (0.68 to 0.99)**	0.05	0.84 (0.67 to 1.05)	0.12
Often no-one is available to look after my child if they cannot go to school ‡	5-point Likert-scale (1=strongly agree, 5=strongly disagree)	N=166, M=3.46, SD=1.44	N= 84, M=3.11, SD=1.55	0.85 (0.71 to 1.02)	0.07	0.88 (0.72 to 1.07)	0.20
My child does not want to take time off school ‡	5-point Likert-scale (1=strongly agree, 5=strongly disagree)	N= 162, M=2.43, SD=1.17	N=83, M=2.11, SD=1.14	**0.78* (0.61 to 0.99)**	0.04	0.82 (0.63 to 1.05)	0.12

For peer review only - http://bmjopen.bmj.com/site/about/guidelines.xhtml

My child makes their own decisions about when they go to school ‡	5-point Likert-scale (1=strongly agree, 5=strongly disagree)	N=166, M=4.25, SD=0.98	N=83, M=3.60, SD=1.50	**0.65** (0.52 to 0.81)**	<0.001	**0.65** (0.50 to 0.83)**	<0.001
My child should go to school if they have taken medication (e.g., Calpol, paracetamol) ‡	5-point Likert-scale (1=strongly agree, 5=strongly disagree)	N=164, M=3.04, SD=1.30	N=81, M=2.25, SD=1.20	**0.60** (0.48 to 0.76)**	<0.001	**0.57** (0.44 to 0.75)**	<0.001
If children have common illnesses (e.g., a cold), they should go to school ‡	5-point Likert-scale (1=strongly agree, 5=strongly disagree)	N=166, M=2.30, SD=1.09	N=83, M=2.13, SD=1.03	0.86 (0.66 to 1.11)	0.24	0.83 (0.63 to 1.09)	0.19
Children build up their immunity by mixing with children who have common illnesses (e.g., a cold) ‡	5-point Likert-scale (1=strongly agree, 5=strongly disagree)	N=164, M=1.93, SD=0.88	N=84, M=1.90, SD=1.03	0.97 (0.73 to 1.29)	0.86	0.92 (0.67 to 1.26)	0.61
Other children with common illnesses (e.g., a cold) go to school ‡	5-point Likert-scale (1=strongly agree, 5=strongly disagree)	N=162, M=1.88, SD=0.79	N=83, M=1.84, SD=0.99	0.96 (0.70 to 1.30)	0.77	0.91 (0.65 to 1.27)	0.58
If children have mild symptoms of an illness, they should go to school ‡	5-point Likert-scale (1=strongly agree, 5=strongly disagree)	N=162, M=1.88, SD=0.79	N=80, M=2.46, SD=1.00	**0.77* (0.59 to 0.99)**	0.04	**0.75* (0.56 to 0.99)**	0.05
Going to school is important for my child's mental health ‡	5-point Likert-scale (1=strongly agree, 5=strongly disagree)	N=164, M=1.45, SD=0.67	N=83, M=1.66, SD=0.93	**1.41* (1.01 to 1.97)**	0.04	1.43 (0.99 to 2.06)	0.06
When my child says they are too ill to attend school, I let them stay at home ‡	5-point Likert-scale (1=strongly agree, 5=strongly disagree)	N=161, M=2.58, SD=0.67	N=84, M=2.56, SD=1.15	0.98 (0.77 to 1.25)	0.87	0.96 (0.74 to 1.25)	0.78

*P≤ 0.05 and formatted bold.
**P≤ 0.001 and formatted bold.
†Odds ratio adjusted by parent gender, age, living region, household income, employment status, and education level.
‡The exact wording used in the survey.
§Working includes students and volunteers.
Abbreviations: N = number of observations; M = mean; SD = standard deviation.

For peer review only

Table 2. Children who engaged in each activity reported on when they had stay-at-home symptoms (n=251)

Exact statement wording, in the order that they were asked to participants.	Children engaged in activity ("non-adherent"), n (%)	Children did not engage activity ("adherent"), n (%)
Went to school	60 (24%)	191 (76%)
Went to a club or lesson outside of school	29 (12%)	222 (88%)
Visited someone from another household	27 (11%)	224 (89%)
Someone from another household visited the child	15 (6%)	236 (94%)
Someone from another household visited our household	24 (10%)	227 (90%)

DISCUSSION

We found that 33% of children were non-adherent to UK Government guidance, such as attending school or clubs or socialising with others when experiencing symptoms where it is advised to stay at home. These findings align with previous reports suggesting that this behaviour, known as presenteeism, is common [12-14]. We also found that non-adherence was more likely for children with younger parents and for specific parental attitudes about symptoms of illness and their children's behaviour. This is concerning because children who attend school and socialise whilst unwell risk spreading their illness to others, increasing the likelihood that other children will need to take time off school and miss out on extra-curricular and social activities, and putting vulnerable people that they come into contact with at risk.

We found that children were significantly more likely to be non-adherent when their parents believed that children should go to school when their symptoms were mild. This finding mirrors Woodland, et al. [12] and Webster, et al. [15] who found people were more likely to attend school or work with an infectious illness when they perceived their symptoms to be mild. We also found that children were more likely to be non-adherent if a parent felt that attendance at school was appropriate if analgesic medication had been taken. These findings suggest that a key consideration is whether the child will cope with school, rather than whether they might spread illness to others. These findings align with previous research that suggested families were less likely to self-isolate with symptoms that may indicate COVID-19 when parents, a) perceived the symptoms to be mild, b) were unsure of the cause of symptoms and c) perceived a reduced severity of symptoms after taking analgesic medication [25]. A greater focus on the importance of decreasing social mixing when symptomatic may help reduce the incidence and spread of infectious disease outbreaks in schools and to others in the community.

Children were significantly more likely to be non-adherent where their parent reported that the child was responsible for making their own decisions about when

For peer review only - http://bmjopen.bmj.com/site/about/guidelines.xhtml

they go to school. This mirrors the suggestion that children are more important in decision-making than previously recognised [12]. Educating children, particularly teenagers who may have a greater say over their attendance, about the need to remain at home when ill may be an important part of any future strategy to reduce presenteeism. But this finding may need to be taken with caution; the data were collected in the autumn term when schools re-opened following national school closures due to the pandemic. Reports show that children were keen to get back to school at this time, which may have impacted this result [26]. Still, educating parents on these issues is also important. We found that parents who were aged 45 years or younger were significantly more likely to have a child that was non-adherent. We can presume that these parents have younger children, and therefore we expect parents to have more control over their child's activities compared to older children. While younger parents may rely on more support from outside the household with childcare, in contrast to previous findings [15]. We did not find a significant association between children who were non-adherent and whether parents had someone available to look after their child if they could not go to school [12,15].

We were surprised that we did not find a significant association between children who were non-adherent and parents who felt their child had missed too much school and were behind in school. This was also replicated in our sensitivity analyses, that focused on children who had attended school and excluded all other types of non-adherent. Webster, et al. [15] found that people who had missed too much work and were concerned about their workload were more likely to attend work with an infectious illness. Woodland, et al. [12] also found that high motivations about school and high school absence were found to increase the risk of school-based presenteeism. We cannot be sure why our results contrast with previous research, but this could be due to the differences between parents not attending work (e.g., financial implications) and children not attending school (e.g., educational impacts) and pandemic-related reasons (e.g., increased access to online schooling and activities). That being said, the adjusted odd ratio is 0.84 (95% confidence interval 0.69 to 1.02), which follows a trend that we expected, it could be that a significant association was not found due to a small sample size.

We suspected that there may have been an association between parents with higher education and children's non-adherence. Research indicates that parents who are educated may place higher importance in their children's education [27]. However, this was not the case, and similar levels of non-adherence were found; 31% for children with parents with a-level or below compared to 36% of children with parents with a degree or above. We suggest that the number of parents who are highly educated may be higher than the target population, which may have had an impact on these findings.

We found no significant associations between gender of the parental participant and reports of a child's adherence.[12] Unfortunately, as children's demographic data were not collected, we were unable to explore associations with this variable in the study or other child demographic characteristics that may have an impact on our findings. For example, it has been suggested that children who are in transition years are at increased risk of school-based presenteeism [12]. Children need to be

For peer review only - http://bmjopen.bmj.com/site/about/guidelines.xhtml

included in future studies about school-based presenteeism as it is common for only the parents to be the participants [12].

There were several limitations to this study. On average 69% of children report at least one episode of school-based presenteeism [12], which is higher than our finding of 33%. However, this average was taken from three studies, two of which measured presenteeism over 12 months whereas ours was assessed for less than 4 months. In addition, we asked parents to report about their child's most severe episode of recent illness and we only reported non-adherence for one child from each household for the four months that we measured. As such, we may have underestimated the prevalence of non-adherence: children who engaged in multiple bouts of non-adherence and households in which multiple children engaged in non-adherence will not have been identified. We also had a relatively small sample size and therefore we may not have detected small effects [28]. Our findings not only report on presenteeism behaviour but also interacting with others while symptomatic, therefore caution must be taken when comparing our findings to studies solely reporting on school-based presenteeism.

In addition, the data were drawn from a non-probability sample. Whether the sample was behaviourally and psychologically representative of the wider population of parents in the UK is unknown [29], although we have no reason to suspect that the associations within the data cannot be generalised [30]. Our participants were mainly of white ethnicity (91%, n=224/246, n=5 were missing data) and we were unable to include ethnicity in our analysis due to small case numbers. Although this is fairly representative of the UK population, we were unable to compare results across different ethnic groups. We did not include child demographics in our analysis due to survey space limitations thus we were unable to identify how children's characteristics may impact non-adherence. Our data collection occurred during a pandemic, with a substantial focus on the importance of remaining home when potentially infectious and after an extended period of home-schooling. How rates and predictors of presenteeism will change as we emerge from the pandemic is unknown.

Conclusion

One-third of children with symptoms that indicated an infectious illness engaged in activities outside the home, including going to school or socialising with others. This behaviour goes against UK Government advice that is in place to prevent the spread of disease. We found that younger parents, children who made their own decisions about school attendance, children who had taken analgesic medication and having mild symptoms were risk factors for non-adherence. We suggest that parents and teenagers may benefit from guidance to help them make informed decisions about school attendance whilst unwell to reduce school presenteeism. Given, our study had a small sample size and was conducted during a pandemic, it is recommended that further research is needed to validate our findings in non-pandemic times and to identify possible interventions that may be generalisable to general circulating and seasonal infections.

For peer review only - http://bmjopen.bmj.com/site/about/guidelines.xhtml

Data availability statement

The dataset and survey questions related to this study can be accessed via the King's College London research data repository, KORDS (DOI 10.18742/19519444). The larger five-wave dataset will be archived with the UK Data Service after the end of the project, reference ES/V015494/1. This dataset can be matched with the full dataset on the ID variable.

Contributors

All authors contributed to the conceptualisation of the study and approved the final draft. LW designed the survey, analysed the data and drafted the manuscript. LES, RA and GJR designed the survey and edited the manuscript. RKW edited the manuscript. RKW, RA and GJR supervised LW.

Funding

This study was funded by the Economic and Social Research Council [grant number ES/P000703/1] and by the National Institute for Health and Care Research Health Protection Research Unit (NIHR HPRU) in Emergency Preparedness and Response [grant number NIHR200890], a partnership between the UK Health Security Agency, King's College London and the University of East Anglia. The views expressed are those of the author(s) and not necessarily those of the NIHR, UKHSA or the Department of Health and Social Care. For the purpose of open access, the author has applied a Creative Commons Attribution (CC BY) licence to any Author Accepted Manuscript version arising. The funders had no role in study design, data collection, data analysis, data interpretation, or writing of the manuscript. The corresponding author had full access to all the data and had final responsibility for the decision to submit for publication.

Competing interests

GJR, RA and LS participate in the UK's Scientific Advisory Group for Emergencies, or its subgroups. These groups did not fund the study or authors. RA is an employee of the UK Health Security Agency. LW and RKW have no competing interests to declare.

For peer review only

REFERENCES

1. National Health Service. Is my child too ill for school? 2021 [updated 1 April. Available from: https://www.nhs.uk/live-well/healthy-body/is-my-child-too-ill-for-school/ accessed 3 February 2022.
2. HM Government. Health protection in schools and other childcare facilities 2021 [updated 3 March 2021. Available from: https://www.gov.uk/government/publications/health-protection-in-schools-and-other-childcare-facilities accessed 9 February 2022.
3. HM Government. Stay at home: guidance for households with possible or confirmed coronavirus (COVID-19) infection 2022 [updated 1 February. Available from: https://www.gov.uk/government/publications/covid-19-stay-at-

For peer review only - http://bmjopen.bmj.com/site/about/guidelines.xhtml

home-guidance/stay-at-home-guidance-for-households-with-possible-coronavirus-covid-19-infection accessed 3 February 2022.
4. HM Government. Prime Minister's statement on coronavirus (COVID-19): 23 March 2020: HM Government,; 2020 [Available from: https://www.gov.uk/government/speeches/pm-address-to-the-nation-on-coronavirus-23-march-2020 accessed 11 June 2020.
5. HM Government. Norovirus outbreaks increasing in England, 2022.
6. Simon AK, Hollander GA, McMichael A. Evolution of the immune system in humans from infancy to old age. *Proceedings of the Royal Society B: Biological Sciences* 2015;282(1821):20143085.
7. Woodland L, Hodson A, Webster RK, et al. A Qualitative Study Evaluating the Factors Affecting Families' Adherence to the First COVID-19 Lockdown in England Using the COM-B Model and TDF. *International Journal of Environmental Research and Public Health* 2022;19(12):7305. doi: 10.3390/ijerph19127305
8. Cleary V, Slaughter R, Heathcock R. An Infection Control Programme in Primary Schools and the Wider Public Health Impact. *Journal of Infection Prevention* 2003;4(5):11-17. doi: 10.1177/175717740300400503
9. Malosh RE, Martin ET, Ortiz JR, et al. The risk of lower respiratory tract infection following influenza virus infection: A systematic and narrative review. *Vaccine* 2018;36(1):141-47.
10. Thomson RM, Henderson HJ, Smith-Palmer A. An outbreak of Salmonella Saintpaul in a Scottish childcare facility: the influence of parental under-reporting. *BMC Infectious Diseases* 2019;19(1):847. doi: https://dx.doi.org/10.1186/s12879-019-4516-z
11. Joseph C, Noah N, White J, et al. A review of outbreaks of infectious disease in schools in England and Wales 1979–88. *Epidemiology and Infection* 1990;105(2):419-34. doi: 10.1017/s0950268800048007
12. Woodland L, Brooks SK, Webster RK, et al. Risk factors for school-based presenteeism in children: a systematic review. *BMC psychology* 2023;11(1):169.
13. Johansen V. Sick and still at school: an empirical study of sickness presence among students in Norwegian secondary school. *BMJ Open* 2015;5(9):e008290. doi: https://dx.doi.org/10.1136/bmjopen-2015-008290
14. BUPA. Bupa report reveals that half of all UK parents send children to school when ill 2010 [updated 26 October. Available from: https://pressreleases.responsesource.com/news/60199/bupa-report-reveals-that-half-of-all-uk-parents-send/ accessed 11 June 2020.
15. Webster RK, Liu R, Karimullina K, et al. A systematic review of infectious illness Presenteeism: prevalence, reasons and risk factors. *BMC Public Health* 2019;19(1):799. doi: 10.1186/s12889-019-7138-x
16. Ipsos MORI. About us n.d [Available from: https://www.ipsos.com/en-uk/about-us accessed 17 February 2022.
17. Ipsos MORI. Ipsos Answers Esomar's Questions to help online research buyers 2023 [updated 30 March. Available from: https://www.ipsos.com/en/ipsos-answers-esomars-questions-help-online-research-buyers accessed 17 July 2023.
18. PAMCo. PAMCo 4 2019 2019 [Available from: https://pamco.co.uk/2019/12/09/pamco-4-2019-data/ accessed 22 July 2023.

For peer review only - http://bmjopen.bmj.com/site/about/guidelines.xhtml

19. Office for National Statistics. Families and the labour market, UK: 2019 2019 [updated 24 October. Available from: https://www.ons.gov.uk/employmentandlabourmarket/peopleinwork/employmentandemployeetypes/articles/familiesandthelabourmarketengland/2019 accessed 10 March 2022.
20. Office for National Statistics. The effects of taxes and benefits on household income, disposable income estimate: financial year ending 2021 2023 [updated 25 January. Available from: https://www.ons.gov.uk/peoplepopulationandcommunity/personalandhouseholdfinances/incomeandwealth/datasets/householddisposableincomeandinequality accessed 22 July 2023.
21. Office for National Statistics. Education, England and Wales: Census 2021 2023 [updated 10 January. Available from: https://www.ons.gov.uk/peoplepopulationandcommunity/educationandchildcare/bulletins/educationenglandandwales/census2021 accessed 4 September 2023.
22. IBM Corp. IBM SPSS Statistics for Windows, 28.0 Armonk, NY 2021 [
23. von Elm E, Altman DG, Egger M, et al. The Strengthening the Reporting of Observational Studies in Epidemiology (STROBE) Statement: Guidelines for Reporting Observational Studies. *Annals of Internal Medicine* 2007;147(8):573-77. doi: 10.7326/0003-4819-147-8-200710160-00010
24. Office for National Statistics. Families and households 2023 [updated 18 May. Available from: https://www.ons.gov.uk/peoplepopulationandcommunity/birthsdeathsandmarriages/families/datasets/familiesandhouseholdsfamiliesandhouseholds accessed 17 July 2023.
25. Woodland L, Mowbray F, Smith LE, et al. What influences whether parents recognise COVID-19 symptoms, request a test and self-isolate: A qualitative study. *PLOS ONE* 2022;17(2):e0263537. doi: 10.1371/journal.pone.0263537
26. Children's Commissioner. Some sort of normal 2020 [updated 2 November. Available from: https://www.childrenscommissioner.gov.uk/education/ accessed 11 March 2022.
27. Woodland L, Smith LE, Webster RK, et al. Why did some parents not send their children back to school following school closures during the COVID-19 pandemic: a cross-sectional survey. *BMJ Paediatrics Open* 2021;5(1):e001014. doi: 10.1136/bmjpo-2020-001014
28. Button KS, Ioannidis JPA, Mokrysz C, et al. Power failure: why small sample size undermines the reliability of neuroscience. *Nature Reviews Neuroscience* 2013;14(5):365-76. doi: 10.1038/nrn3475
29. Epstein WM. Response bias in opinion polls and American social welfare. *The Social Science Journal* 2006;43(1):99-110. doi: 10.1016/j.soscij.2005.12.010
30. Kohler U. Possible Uses of Nonprobability Sampling for the Social Sciences. *Survey Methods: Insights from the Field* 2019 [updated 2 April. Available from: https://surveyinsights.org/?p=10981 accessed 29 July 2020.

For peer review only

Text S1: Survey questions

Standard demographic variables:

1. What is your date of birth?

1. YEAR
2. 1910 1910
3. ...
4. 2015 2015
5. MONTH
6. January
7. February
8. March
9. April
10. May
11. June
12. July
13. August
14. September
15. October
16. November
17. December

2. Which of the following describes how you think of yourself?

1. Male
2. Female
3. In another way
4. Prefer not to answer

3. Where do you live? Please note: This question may be considered personal. We would like to remind you that your participation is strictly voluntary and that your responses are used for research purposes only. The answers that you provide will be presented in aggregate form and none of them will be linked back to you in any way. All data will be collected and processed in adherence to the Market Research Society's Code of Conduct and the General Data Protection Regulation (GDPR).

1. Postcode
2. Postal Town
3. Prefer Not to Answer

4. Where do you live?

1. North East
2. North West
3. Yorkshire and The Humber
4. West Midlands
5. East Midlands
6. East of England

For peer review only

7. South West
8. South East
9. Greater London
10. Wales
11. Scotland
12. Northern Ireland

5. How many people are living or staying at your current address? (Include yourself and any other adults or children who are currently living or staying at this address for at least two months)

1. 1
2. 2
3. 3
4. 4
5. 5
6. 6
7. 7
8. 8
9. 9
10. 10
11. 11
12. 12+

6. How many children under the age of 18 are living in your household? Please reference only the children for which you are the parent or legal guardian. (If there are no children under 18 in your household, please type 0)

7. Please provide us with the following information about the children under the age of 18 in your household. Please reference only the children for which you are the parent or legal guardian.

1. Relationship
2. Parent (biological or adopted)
3. Legal guardian
4. Other (e.g. step-child)
5. Gender
6. Boy
7. Girl
8. Year of birth
9. Month of birth

8. What is your highest level of education attained?

1. Primary school
2. Secondary school (age under 15 years old)
3. GNVQ / GSVQ / GCSE/ SCE standard.
4. NVQ1, NVQ2
5. NVQ3/ SCE Higher Grade/ Advanced GNVQ/ GCE A/AS or similar.
6. NVQ4 / HNC / HND / Bachelor's degree or similar.
7. NVQ5 or post-graduate diploma.

For peer review only

For peer review only - http://bmjopen.bmj.com/site/about/guidelines.xhtml

9. The next question may be considered personal, but it is not mandatory to answer. If you do, we assure you that your responses will be kept strictly confidential and used for research purposes only. What is the COMBINED TOTAL ANNUAL INCOME (pre-tax) earned by all members of your household? Please include all your income sources : salaries, scholarships, pension and Social Security benefits, dividends from shares, income from rental properties, child support and alimony etc.

1. Under £5,000
2. £5,000 - £9,999
3. £10,000 - £14,999
4. £15,000 - £19,999
5. £20,000 - £24,999
6. £25,000 - £34,999
7. £35,000 - £44,999
8. £45,000 - £54,999
9. £55,000 - £99,999
10. £100,000 or more
11. Prefer not to answer

10. What is your current employment status?

1. Employed full-time
2. Employed part-time
3. Self employed
4. Unemployed but looking for a job
5. Unemployed and not looking for a job/Long-term sick or disabled
6. Full-time parent, homemaker
7. Retired
8. Student/Pupil

11. In which of the below categories does your occupation fall? If retired or unemployed, please indicate the category closest to your previous occupation.

Responses copied from https://www.ilo.org/public/english/bureau/stat/isco/isco88/

12. What is the occupation of the person with the highest income? If retired or unemployed, please indicate the category closest to his/her previous occupation.

Responses copied from https://www.ilo.org/public/english/bureau/stat/isco/isco88/

In this survey, some questions will be about health and perceptions about, coronavirus, politics, religious beliefs, etc. A "Prefer not to answer" option will be available for you to select, if the case. Ipsos is running the survey and collecting such data in order to understand attitudes towards public health measures in the context of the coronavirus pandemic, and trust in their efficacy.

Ipsos has been commissioned by the University of Bristol and King's College London to carry out this research. Information collected through the survey will be made available to the team of researchers at the University of Bristol and King's College London in an anonymised format, unless you provide your permission to pass personal data to them. At

For peer review only - http://bmjopen.bmj.com/site/about/guidelines.xhtml

no point will any information that would allow you to be identified be made available to the public.

Participation is completely voluntary and you may withdraw your consent at any time. Your survey answers will be combined with the answers from all other participants and used for social research purposes only, and your personal data will be held for no longer than 12 months within Ipsos and no longer than April 2023 from researchers at the University of Bristol and King's College London.

Do you accept the collection of personal information and of health and perceptions about coronavirus related data?

1. *Yes, I accept*
2. *No, I do not accept*

13. Which of these applies to your home?

1. Being bought on a mortgage
2. Owned outright by household
3. Rented from a local authority
4. Rented from a housing association/trust
5. Rented from a private landlord
6. Other
7. Refused/don't know

The next question may be considered personal, but it is not mandatory to answer. If you do, we assure you that your responses will be kept strictly confidential and used for research purposes only.

14. What is your ethnic group?

Please select only one

White

1. English / Welsh / Scottish / Northern Irish / British
2. Irish
3. Gypsy or Irish Traveller
4. Any other White background

Mixed / multiple ethnic groups

5. White and Black Caribbean
6. White and Black African
7. White and Asian
8. Any other Mixed / multiple ethnic background

Asian / Asian British

9. Indian
10. Pakistani

For peer review only - http://bmjopen.bmj.com/site/about/guidelines.xhtml

11. Bangladeshi
12. Chinese
13. Any other Asian background

Black / African / Caribbean / Black British

14. African
15. Caribbean
16. Any other Black / African / Caribbean background

Other ethnic group

17. Arab
18. Any other ethnic group, please write in
19. Prefer not to answer

For peer review only

Survey questions

For the next set of questions, we would like you to think about your [number of children] youngest children who usually attend school.

15. Since *about* [Scotland only] the start of the school year (September 2021), please tick, for each child, any new symptoms that they developed for any reason (e.g. a cold, allergic reaction, coronavirus, etc.), even if some of the symptoms were mild. If they have developed new symptoms more than once during that period (for example if they have had a cold in one week, and an allergic reaction a couple of weeks later), please just tell us about the most severe set of symptoms that they had (for example, tell us about the cold, or the allergic reaction, whichever seemed worse). Please tick any that apply.

1. New, continuous cough
2. High temperature / fever
3. Diarrhoea
4. Nausea / feeling sick
5. Vomiting / being sick
6. Earache
7. A rash
8. Loss of sense of smell (fully or partial)
9. Loss of taste
10. Sore throat
11. Headache
12. Muscle ache
13. Breathlessness, tight chest, or wheezing
14. Feeling tired or exhausted
15. I don't have any (more) children who usually attend school
16. None of these
17. Don't know
18. Prefer not to say

For peer review only - http://bmjopen.bmj.com/site/about/guidelines.xhtml

1. Child 1 (youngest)
2. Child 2
3. Child 3
4. Child (oldest)

16. Please think about your child who most recently developed symptoms. When they had symptoms, did they do any of the following?
Please tick any that apply. Do not count things that they did online.

1. Went to school
2. Went to a club or lesson outside of school
3. Visited someone from another household
4. Someone from another household visited the child
5. Someone from another household visited our household
6. Took a lateral flow test (LFT) to see whether they had coronavirus (this is a rapid test that gives results in 30 minutes)
7. Took a polymerase chain reaction (PCR) test to see whether they had coronavirus (this is a test that is sent off to a laboratory)
8. Took both a lateral flow test (LFT, a rapid test that gives results in 30 minutes) and a polymerase chain reaction (PCR) test (a test that you send off to the laboratory) to see whether they had coronavirus
9. Took a test to see whether they had coronavirus, but I am not sure what type of test it was
10. None of these
11. Prefer not to say

17. Please still think about your child who most recently developed symptoms. You said your child had taken at least one rapid lateral flow test (LFT). Were any of the results positive?

1. Yes, this child had one or more rapid lateral flow tests (LFT) and had a positive result
2. None of the rapid lateral flow tests (LFT) for this child had a positive result
3. Don't know
4. Prefer not to say

18. Please still think about your child who most recently developed symptoms. You said your child had taken a polymerase chain reaction (PCR) test to see whether they had coronavirus. What was the result?

1. My child tested positive
2. My child tested negative
3. The result was inconclusive
4. I have not received the results
5. Don't know
6. Prefer not to say

For peer review only

For peer review only - http://bmjopen.bmj.com/site/about/guidelines.xhtml

19. Thinking about the same child who most recently developed symptoms, to what extent, if at all, do you agree or disagree with the following statements about this child, as a result of having these symptoms?

1. My child has missed too much school since September this year
2. My child is behind at school
3. My child often says they have symptoms of illnesses when they do not
4. Often no-one is available to look after my child if they cannot go to school
5. My child does not want to take time off school
6. My child makes their own decisions about when they go to school
7. My child should go to school if they have taken medication (e.g., Calpol, paracetamol)

    1. Strongly agree
    2. Tend to agree
    3. Neither agree nor disagree
    4. Tend to disagree
    5. Strongly disagree
    6. Don't know
    7. Prefer not to say

20. Now thinking in general, to what extent, if at all, do you agree or disagree with the following statements?

1. If children have common illnesses (e.g., a cold), they should go to school
2. Children build up their immunity by mixing with children who have common illnesses (e.g., a cold)
3. Other children with common illnesses (e.g. a cold) go to school
4. If children have mild symptoms of an illness, they should go to school
5. Going to school is important for my child's mental health
6. When my child says they are too ill to attend school, I let them stay at home

    1. Strongly agree
    2. Tend to agree
    3. Neither agree nor disagree
    4. Tend to disagree
    5. Strongly disagree
    6. Don't know
    7. Prefer not to say

For peer review only - http://bmjopen.bmj.com/site/about/guidelines.xhtml

Supplement 3: Checklist for Reporting Of Survey Studies (CROSS)

Section/topic	Item	Item description	Reported on page #
**Title and abstract**			
Title and abstract	1a	State the word "survey" along with a commonly used term in title or abstract to introduce the study's design. #2	
	1b	Provide an informative summary in the abstract, covering background, objectives, methods, findings/results, interpretation/discussion, and conclusions. #2	
**Introduction**			
Background	2	Provide a background about the rationale of study, what has been previously done, and why this survey is needed. #4	
Purpose/aim	3	Identify specific purposes, aims, goals, or objectives of the study. #4	
**Methods**			
Study design	4	Specify the study design in the methods section with a commonly used term (e.g., cross-sectional or longitudinal). #5	
Data collection methods	5a	Describe the questionnaire (e.g., number of sections, number of questions, number and names of instruments used).	
	5b	Describe all questionnaire instruments that were used in the survey to measure #5 particular concepts. Report target population, reported validity and reliability information, scoring/classification procedure, and reference links (if any). #5	
	5c	Provide information on pretesting of the questionnaire, if performed (in the article or in an online supplement). Report the method of pretesting, number of times questionnaire was pre-tested, number and demographics of participants used for pretesting, and the level of similarity of demographics between pre-testing participants and sample population. #5	
	5d	Questionnaire if possible, should be fully provided (in the article, or as appendices or as an online supplement). #this has been provided (Text S1 Survey)	
Sample characteristics	6a	Describe the study population (i.e., background, locations, eligibility criteria for participant inclusion in survey, exclusion criteria). #5	
	6b	Describe the sampling techniques used (e.g., single stage or multistage sampling, simple random sampling, stratified sampling, cluster sampling, convenience sampling). Specify the locations of sample participants whenever clustered sampling was applied. #5	
	6c	Provide information on sample size, along with details of sample size calculation.	
	6d	Describe how representative the sample is of the study population (or target population if possible), particularly for population-based surveys. #5	

For peer review only

Survey administration	7a	Provide information on modes of questionnaire administration, including the type and number of contacts, the location where the survey was conducted (e.g., outpatient room or by use of online tools, such as SurveyMonkey). #5
	7b	Provide information of survey's time frame, such as periods of recruitment, exposure, and follow-up days. #5
	7c	Provide information on the entry process: >For non-web-based surveys, provide approaches to minimize human error in data entry. >For web-based surveys, provide approaches to prevent "multiple participation" of participants. #5
Study preparation	8	Describe any preparation process before conducting the survey (e.g., interviewers' training process, advertising the survey). #5
Ethical considerations	9a	Provide information on ethical approval for the survey if obtained, including informed consent, institutional review board [IRB] approval, Helsinki declaration, and good clinical practice [GCP] declaration (as appropriate). #7
	9b	Provide information about survey anonymity and confidentiality and describe what mechanisms were used to protect unauthorized access. #7
Statistical analysis	10a	Describe statistical methods and analytical approach. Report the statistical software that was used for data analysis. #7
	10b	Report any modification of variables used in the analysis, along with reference (if available). #7
	10c	Report details about how missing data was handled. Include rate of missing items, missing data mechanism (i.e., missing completely at random [MCAR], missing at random [MAR] or missing not at random [MNAR]) and methods used to deal with missing data (e.g., multiple imputation). #7
	10d	State how non-response error was addressed. #5
	10e	For longitudinal surveys, state how loss to follow-up was addressed.
	10f	Indicate whether any methods such as weighting of items or propensity scores have been used to adjust for non-representativeness of the sample. #5
	10g	Describe any sensitivity analysis conducted. #7
**Results**		
Respondent characteristics	11a	Report numbers of individuals at each stage of the study. Consider using a flow diagram, if possible. #8
	11b	Provide reasons for non-participation at each stage, if possible. #8
	11c	Report response rate, present the definition of response rate or the formula used to calculate response rate. #NA
	11d	Provide information to define how unique visitors are determined. Report number of unique visitors along with relevant proportions (e.g., view proportion, participation proportion, completion proportion). #NA
Descriptive results	12	Provide characteristics of study participants, as well as information on potential confounders and assessed outcomes. #8

For peer review only

Main findings	13a	Give unadjusted estimates and, if applicable, confounder-adjusted estimates along with 95% confidence intervals and p-values. #10
	13b	For multivariable analysis, provide information on the model building process, model fit statistics, and model assumptions (as appropriate). #8
	13c	Provide details about any sensitivity analysis performed. If there are considerable amount of missing data, report sensitivity analyses comparing the results of complete cases with that of the imputed dataset (if possible). #NA
**Discussion**		
Limitations	14	Discuss the limitations of the study, considering sources of potential biases and imprecisions, such as non-representativeness of sample, study design, important uncontrolled confounders. #12
Interpretations	15	Give a cautious overall interpretation of results, based on potential biases and imprecisions and suggest areas for future research. #13
Generalizability	16	Discuss the external validity of the results. #13
**Other sections**		
Role of funding source	17	State whether any funding organization has had any roles in the survey's design, implementation, and analysis. #14
Conflict of interest	18	Declare any potential conflict of interest. #15
Acknowledgements	19	Provide names of organizations/persons that are acknowledged along with their contribution to the research. #14

For peer review only

Reporting checklist for cross sectional study.

Based on the STROBE cross sectional guidelines.

Instructions to authors

Complete this checklist by entering the page numbers from your manuscript where readers will find each of the items listed below.

Your article may not currently address all the items on the checklist. Please modify your text to include the missing information. If you are certain that an item does not apply, please write "n/a" and provide a short explanation.

Upload your completed checklist as an extra file when you submit to a journal.

In your methods section, say that you used the STROBE cross sectionalreporting guidelines, and cite them as:

von Elm E, Altman DG, Egger M, Pocock SJ, Gotzsche PC, Vandenbroucke JP. The Strengthening the Reporting of Observational Studies in Epidemiology (STROBE) Statement: guidelines for reporting observational studies.

		Reporting Item	Page Number
**Title and abstract**			
Title	#1a	Indicate the study's design with a commonly used term in the title or the abstract	1

For peer review only

Abstract	#1b	Provide in the abstract an informative and balanced summary of what was done and what was found	2
**Introduction**			
Background / rationale	#2	Explain the scientific background and rationale for the investigation being reported	4
Objectives	#3	State specific objectives, including any prespecified hypotheses	4
**Methods**			
Study design	#4	Present key elements of study design early in the paper	5
Setting	#5	Describe the setting, locations, and relevant dates, including periods of recruitment, exposure, follow-up, and data collection	5
Eligibility criteria	#6a	Give the eligibility criteria, and the sources and methods of selection of participants.	5
	#7	Clearly define all outcomes, exposures, predictors, potential confounders, and effect modifiers. Give diagnostic criteria, if applicable	5
Data sources / measurement	#8	For each variable of interest give sources of data and details of methods of assessment (measurement). Describe comparability of assessment methods if there is more than one group. Give information separately for for exposed and unexposed groups if applicable.	5,6

For peer review only

Bias	#9	Describe any efforts to address potential sources of bias	5
Study size	#10	Explain how the study size was arrived at	5
Quantitative variables	#11	Explain how quantitative variables were handled in the analyses. If applicable, describe which groupings were chosen, and why	6
Statistical methods	#12a	Describe all statistical methods, including those used to control for confounding	7
Statistical methods	#12b	Describe any methods used to examine subgroups and interactions	6
Statistical methods	#12c	Explain how missing data were addressed	7
Statistical methods	#12d	If applicable, describe analytical methods taking account of sampling strategy	n/a
Statistical methods	#12e	Describe any sensitivity analyses	7
**Results**			
Participants	#13a	Report numbers of individuals at each stage of study—eg numbers potentially eligible, examined for eligibility, confirmed eligible, included in the study, completing follow-up, and analysed. Give information separately for for exposed and unexposed groups if applicable.	8
Participants	#13b	Give reasons for non-participation at each stage	8

For peer review only

Participants	#13c	Consider use of a flow diagram	n/a
Descriptive data	#14a	Give characteristics of study participants (eg demographic, clinical, social) and information on exposures and potential confounders. Give information separately for exposed and unexposed groups if applicable.	8
Descriptive data	#14b	Indicate number of participants with missing data for each variable of interest	10,11
Outcome data	#15	Report numbers of outcome events or summary measures. Give information separately for exposed and unexposed groups if applicable.	10,11
Main results	#16a	Give unadjusted estimates and, if applicable, confounder-adjusted estimates and their precision (eg, 95% confidence interval). Make clear which confounders were adjusted for and why they were included	10,11
Main results	#16b	Report category boundaries when continuous variables were categorized	10,11
Main results	#16c	If relevant, consider translating estimates of relative risk into absolute risk for a meaningful time period	n/a
Other analyses	#17	Report other analyses done—e.g., analyses of subgroups and interactions, and sensitivity analyses	9
**Discussion**			
Key results	#18	Summarise key results with reference to study objectives	13

For peer review only

Limitations	#19	Discuss limitations of the study, taking into account sources of potential bias or imprecision. Discuss both direction and magnitude of any potential bias.	14,15
Interpretation	#20	Give a cautious overall interpretation considering objectives, limitations, multiplicity of analyses, results from similar studies, and other relevant evidence.	14,15
Generalisability	#21	Discuss the generalisability (external validity) of the study results	14,15
**Other Information**			
Funding	#22	Give the source of funding and the role of the funders for the present study and, if applicable, for the original study on which the present article is based	15

The STROBE checklist is distributed under the terms of the Creative Commons Attribution License CC-BY. This checklist was completed on 03. January 2023 using https://www.goodreports.org/, a tool made by the EQUATOR Network in collaboration with Penelope.ai

For peer review only
